# High-grade Gliomas Exhibit Higher Peritumoral Fractional Anisotropy and Lower Mean Diffusivity than Intracranial Metastases

**DOI:** 10.3389/fsurg.2017.00018

**Published:** 2017-04-10

**Authors:** Kevin S. Holly, Benjamin J. Barker, Derrick Murcia, Rebekah Bennett, Piyush Kalakoti, Christina Ledbetter, Eduardo Gonzalez-Toledo, Anil Nanda, Hai Sun

**Affiliations:** ^1^Department of Neurosurgery, Louisiana State University Health Sciences Center Shreveport, Shreveport, LA, USA; ^2^Department of Neurology, Louisiana State University Health Sciences Center Shreveport, Shreveport, LA, USA; ^3^Department of Biological Sciences, Louisiana State University Shreveport, Shreveport, LA, USA; ^4^Department of Radiology, Louisiana State University Health Sciences Center Shreveport, Shreveport, LA, USA

**Keywords:** glioma, metastases, diffusion tensor imaging, peritumoral, fractional anisotropy, fluid-attenuated inversion recovery, mean diffusivity

## Abstract

Differentiating high-grade gliomas and intracranial metastases through non-invasive imaging has been challenging. Here, we retrospectively compared both intratumoral and peritumoral fractional anisotropy (FA), mean diffusivity (MD), and fluid-attenuated inversion recovery (FLAIR) measurements between high-grade gliomas and metastases. Two methods were utilized to select peritumoral region of interest (ROI). The first method utilized the manual placement of four ROIs adjacent to the lesion. The second method utilized a semiautomated and proprietary MATLAB script to generate an ROI encompassing the entire tumor. The average peritumoral FA, MD, and FLAIR values were determined within the ROIs for both methods. Forty patients with high-grade gliomas and 44 with metastases were enrolled in this study. Thirty-five patients with high-grade glioma and 30 patients with metastases had FLAIR images. There was no significant difference in age, gender, or race between the two patient groups. The high-grade gliomas had a significantly higher tumor-to-brain area ratio compared to the metastases. There were no differences in average intratumoral FA, MD, and FLAIR values between the two groups. Both the manual sample method and the semiautomated peritumoral ring method resulted in significantly higher peritumoral FA and significantly lower peritumoral MD in high-grade gliomas compared to metastases (*p* < 0.05). No significant difference was found in FLAIR values between the two groups peritumorally. Receiver operating curve analysis revealed FA to be a more sensitive and specific metric to differentiate high-grade gliomas and metastases than MD. The differences in the peritumoral FA and MD values between high-grade gliomas and metastases seemed due to the infiltration of glioma to the surrounding brain parenchyma.

## Introduction

Despite high-grade gliomas and metastases being the most commonly encountered intracranial lesions, their differentiation using common non-invasive imaging techniques such as conventional magnetic resonance (MR) sequences and computerized tomography scans is often inconclusive (1). Currently, differentiation relies on correlating clinical history with biopsy, or in case of metastases, searching for primary lesions throughout the body (2).

Measurements computed from diffusion tensor imaging (DTI) such as fractional anisotropy (FA) and mean diffusivity (MD) have been applied to several studies that involve pathological changes within brain tumors (3–7). Previous studies have shown there is no significant difference in intratumoral tensor measurements between gliomas and metastases (4, 6, 7), although one study found FA to be higher in glioblastomas when compared to metastases (5), and another study found apparent diffusion coefficient (ADC) within the tumors to be lower in gliomas than metastases (3).

Diffusion tensor imaging-derived measurements, such as FA and MD, describe the microstructural properties of individual voxels. Anisotropy describes the tendency of water to travel along a single axis. High FA values are expected in white matter tracts that move along a single axis, while low FA values are expected in free water areas such as ventricles. Generally speaking, MD values are inversely correlated to FA values. High MD is expected in voxels with low anisotropy. From a clinical standpoint, FA values are lower and MD values are higher in damaged white matter when compared to healthy tissue. Depending on the specific condition, this is thought to be due to edema, axonal disruption, or a combination of the two (8, 9).

Some studies have investigated peritumoral regions to discern between tumor types (1, 4, 5, 10–12). In contrast to metastatic lesions, the vasogenic edema surrounding gliomas is characterized by infiltrating tumor cells (6, 10, 13–18). Despite this pathological difference, there have been mixed results using DTI metrics in the peritumoral region to differentiate between these two distinct tumor types (19). Although the majority of previous studies demonstrated no difference in the peritumoral FA across gliomas and metastases (6, 10, 11, 14, 20–23), few depicted higher FA in gliomas (11, 19, 24), while others found metastases to have higher FA (17, 25). Most could not find a significant difference in MD or ADC between gliomas and metastatic lesions within the peritumoral region (4, 11, 14, 15, 17, 19, 21, 24, 25). While others found that high-grade gliomas had significantly lower peritumoral MD values in comparison to the metastases (3, 10, 11, 16, 22), one study revealed higher MD in high-grade gliomas compared to metastatic tumors (26).

These conflicting results are possibly due to differences in region of interest (ROI) selection technique and/or small sample sizes. Some studies used subjective placement of ROIs surrounding the tumor to examine FA and/or MD (3, 10, 12, 15, 16, 21, 23, 25–28). Several studies have employed experts to manually draw a perimeter around the tumor (4–7, 11, 14, 19, 22, 24, 29). In addition, the majority of previous studies were limited by small numbers of patients included in the studies (3–7, 10, 11, 14–16, 21, 22, 24–26, 28, 29).

Here, we retrospectively compared both intratumoral and peritumoral FA, MD, and fluid-attenuated inversion recovery (FLAIR) measurements between high-grade gliomas and metastases using a large institutional cohort. Two methods were utilized to select peritumoral ROI. The first method utilized the manual placement of four ROIs adjacent to the lesion. The second method utilized a semiautomated and proprietary MATLAB script to generate a ROI encompassing the entire tumor.

## Materials and Methods

### Study Protocol and Patient Population

Adult patients (>18 years of age) with complete information on MR sequences including DTI and conventional T1- or T1-contrast-enhanced scans without any evidence of movement artifacts and a positive histopathological diagnosis were included. From an initial list of 1,102 tumor surgery patients, 914 were excluded for a diagnosis other than glioma or metastases, or had no record of preoperative DTI and T1 scans. Furthermore, patients with neighboring or bilateral tumors and those having a previous history of neurosurgical intervention or chemotherapy and/or radiotherapy for intracranial tumors were excluded. Midline tumors and those with ventricular extension were excluded as well. A total of 84 patients met the inclusion criteria, with 40 glioma and 44 metastatic lesion patients. Data collected on eligible patients included age, gender, race, and tumor laterality and lobe location.

### Image Acquisition and Preprocessing

All retrieved MRI scans were performed on a 1.5 T clinical MRI systems (GE Medical Systems, Milwaukee, WI, USA). The MR imaging examination included a conventional or contrast-enhanced T1-weighted sequence (TR/TE 9.644/3.82, 256 × 256 matrix size, 1.2-mm slice thickness), diffusion tensor sequence (TR/TE 6,200/103.7, 256 × 256 matrix size, 5-mm slice thickness), and three-dimensional sagittal FLAIR sequence (TR/TE 6,000/129.537, 256 × 256 matrix size, 1.8-mm slice thickness). Retrieved images for eligible patients were converted from DICOM to NRRD format with 3D Slicer version 4.1.1 (http://www.slicer.org) (30). Using 3D Slicer, FA and MD maps were derived from DTI scans and T1, contrast-enhanced T1, and T2-FLAIR scans were registered to the baseline DTI volume (see Data Sheet S1 in Supplementary Material for further details). Subsequently, these images were analyzed using two techniques, a manual sample method and a novel peritumoral ring method.

### Manual Sample Method

**Figure 1:**
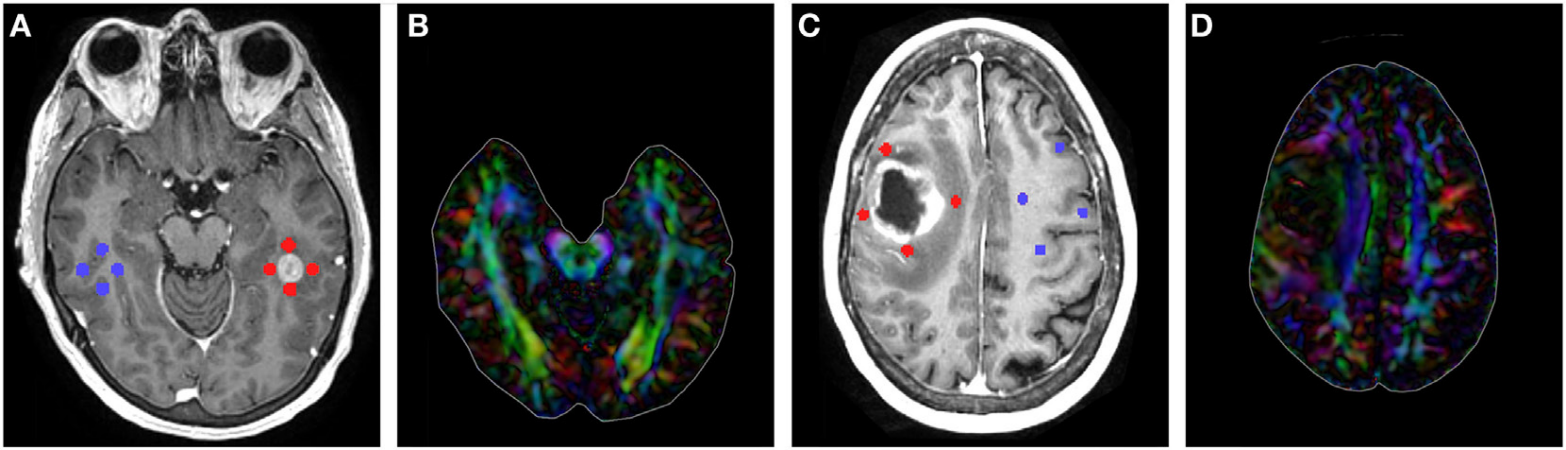
**(A)** T1-contrast scan of a metastatic patient with four peritumoral regions of interest (ROIs) (red) and their contralateral counterparts (blue). **(B)** Diffusion tensor imaging (DTI) color map of the metastatic patient. **(C)** T1-contrast scan of a glioma patient with four peritumoral ROIs (red) and their contralateral counterparts (blue). **(D)** DTI color map of the glioma patient.

Region of interest placement was determined using contrast-enhanced T1. Using 3D Slicer, all ROIs were placed on the slice with the largest tumor area (Figure 1). Four 3-mm ROIs were manually placed in an orthogonal orientation adjacent to the contrast-enhanced region to measure the peritumoral FA and MD. Likewise, four additional 3-mm ROIs were manually placed in the contralateral hemisphere, mirroring the placement of the lesion ROIs. ROIs were carefully placed to avoid sampling of skull or ventricles (see Data Sheet S1 in Supplementary Material for further details) (10, 18, 26). Mean values were calculated across the four ROIs for the affected and contralateral hemispheres for each patient.

### Peritumoral Ring Method

The T1, FA, and MD maps used in the manual sample were exported from 3D Slicer as NIfTI (.nii) files. If FLAIR image sequences were available (*n* = 65), NIfTI files of FLAIR sequences were also registered within 3D slicer and exported. Using a custom MATLAB script, the exact image slices used in the manual sample method were recalled and the average FA, MD, and FLAIR values were determined within the tumor and within the peritumoral region. The ROIs were determined in the DTI and FLAIR images with the guide of the T1 images.

**Figure 2:**
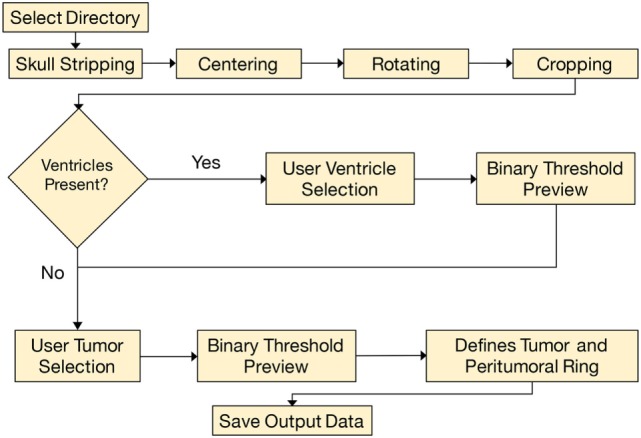
**This is a simplified flowchart of our custom MATLAB script**.

**Figure 3:**
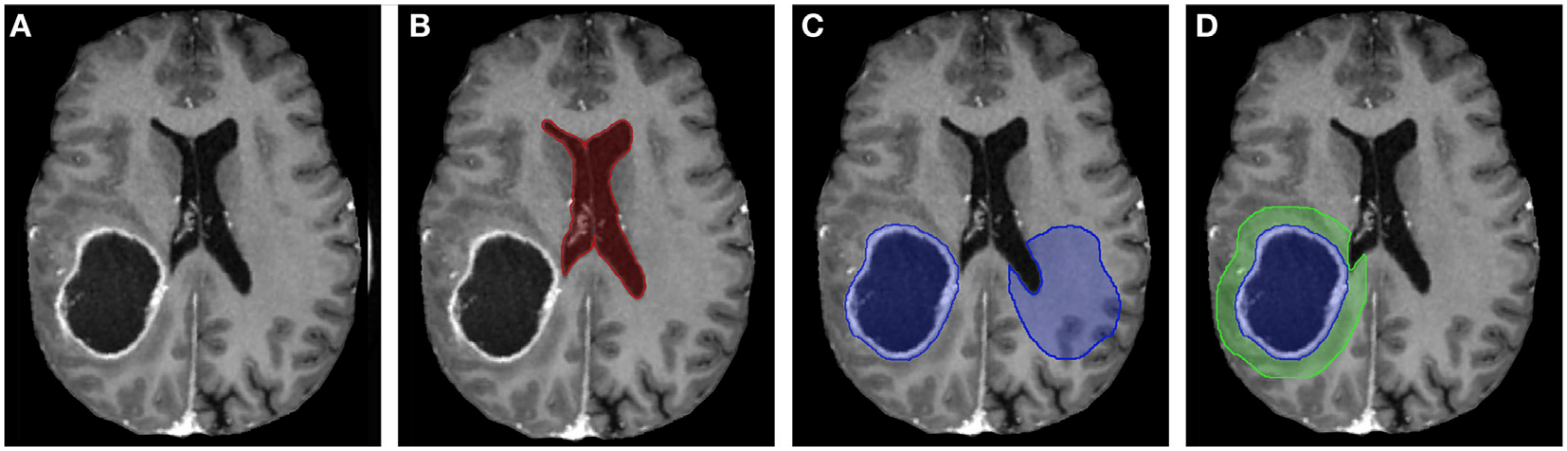
**(A)** T1-contrast scan of a glioma patient. **(B)** A generated ventricle mask (red). **(C)** A generated tumor mask (blue, left) and its mirrored contralateral mask (blue, right). Notice the mirrored mask on the contralateral side does not include the ventricles. **(D)** The expanding peritumoral contour regions of interest (green) surrounding the tumor mask (blue).

The analysis began by tracing the perimeter of the brain within the T1 image slice (Figure 2). The skull was stripped from the image, and two points along the midline of the brain were selected for orientation. The brain image was then rotated, centered, and cropped (Figure 3A). The aligned tensor and FLAIR images were also skull stripped, rotated, centered, and cropped in the exact manner as the T1 image. If ventricles were present within the image slice, the region containing the ventricles were manually selected and refined by a binary threshold (Figure 3B). Similar to the ventricle selection, a binary mask of the tumor region was generated using a binary threshold, which excludes voxels from the ventricles, skull, and the contralateral hemisphere (Figure 3C). The program automatically established a 24 voxel wide peritumoral ring mask (Figure 3D). Mean measurements of the FA, MD, and FLAIR values within the tumor and within the peritumoral ring as well as their contralateral counterparts were calculated (see Data Sheet S1 in Supplementary Material for further details).

### Statistical Analysis

Patient demographics and tumor characteristics across the two tumor groups, viz., high-grade gliomas and metastatic lesions were compared. Categorical variables are reported as frequencies and proportions, and compared using the Pearson χ^2^ test or Fisher’s exact test as appropriate. For evaluating differences in the means across gliomas and metastasis for FA, MD, and FLAIR at intratumoral and peritumoral locations, an independent sample *t*-test was employed (α = 0.05). The independent sample *t*-test was also used to compare means between the ipsilateral ROIs to their contralateral counterparts for FA, MD, and FLAIR. This was performed for both methods (manual sample and peritumoral ring). Boxplots were created for intratumoral and peritumoral FA, MD, and FLAIR that display the median (horizontal line) and the interquartile range (IQR) (box). Data points beyond the whiskers (1.5 × IQR) were considered outliers (circles), and extreme cases (beyond 3 × IQR) were denoted as stars. These data points were not excluded from the statistical analysis. All statistical tests were 2-tailed, and all analyses were performed using SPSS version 24.0 (IBM, Armonk, NY, USA).

We tested three different prediction models. The first assumes the diagnosis is metastasis if the FA is below a specific threshold. The second assumes the diagnosis is metastasis if the MD is greater than a specific threshold. The third was based on the combination of both FA and MD thresholds. The accuracy, sensitivity, and specificity of each prediction model compared to the true diagnosis were determined along with the area under the curve (AUC). Binomial proportion confidence intervals for the accuracy, sensitivity, and specificity were calculated using normal approximation interval (Wald interval) since the sample size (*n* = 84) was greater than 30 and the proportions were not close to 0 or 1 (31). The AUC was approximated by the simple trapezoidal method as shown in Eq. 1 (32, 33):

(1) $$\text{AUC}=\frac{\text{sensitivity}+\text{specificity}}{2}.$$

The receiver operating curve (ROC) curves were generated with data from a custom script written in MATLAB to justify the FA and MD threshold selections. In the ROC curves, the most optimal threshold would be located in the top left of the graph as this is where sensitivity and specificity are the highest. The FA and MD threshold values that provided the maximum AUC along with a reasonable sensitivity and specificity ratio would be the most optimal.

## Results

A total of 84 patients from Louisiana State University Health Sciences Center’s database met the inclusion criteria. Included were 40 glioma patients and 44 metastatic patients. Sixty-five patients had FLAIR images (35 high-grade glioma and 30 metastases). The origins of metastatic lesions were 32 lungs, 6 breasts, 2 lymphomas, 1 colon, 1 melanoma, and 1 uterine. The highest number of brain metastases originated in the lung, which is reflective in population studies (34, 35). Table 1 provides the patient demographics and tumor imaging characteristics. There was no significant difference in age, gender, or race between the two patient groups. The high-grade gliomas had a significantly higher tumor-to-brain area ratio compared to the metastases. In our study, the high-grade gliomas were more likely to be located in the parietal and temporal lobes (*p* = 0.027; *p* = 0.044), whereas the metastatic lesions were more likely to be located in the frontal lobe (*p* = 0.008). For the 2D slices analyzed, the average ROI area of the tumors, peritumoral rings, and manual samples were 787 ± 637, 2,305 ± 606, and 70 ± 23 (SD) pixels, respectively.

**Table 1:** **Patient demographics and tumor characteristics**.

Characteristics	High-grade gliomas (*n* = 40)	Metastatic lesions (*n* = 44)	Overall (*n* = 88)	*p* value
**Age, in years**				
Mean age ± SD	56.9 ± 13.9	57.5 ± 10.7	57.2 ± 12.2	0.644
Range	26–79	30–82	26–82	
**Gender, *n* (%)**				
F	14 (35.0)	22 (50.0)	36 (42.9)	0.165
M	26 (65.0)	22 (50.0)	48 (57.1)	
**Race, *n* (%)**				
Whites	29 (72.5)	24 (54.5)	53 (63.1)	0.089
Others	11 (27.5)	20 (45.5)	31 (36.9)	
**Tumor laterality, *n* (%)**				
Right	18 (45.0)	23 (47.7)	41 (48.8)	0.505
Left	22 (55.0)	21 (52.3)	43 (51.2)	
**Lobes, *n* (%)**				
Parietal	16 (40.0)	8 (18.2)	24 (28.6)	0.027
Frontal	8 (20.0)	21 (47.7)	29 (34.5)	0.008
Temporal	14 (35.0)	7 (15.9)	21 (25.0)	0.044
Occipital	2 (5.0)	8 (18.2)	10 (11.9)	0.062
**Tumor-to-brain area ratio (mean ± SD)**	0.067 ± 0.03	0.040 ± 0.04	0.053 ± 0.04	**<**0.001

**Figure 4:**
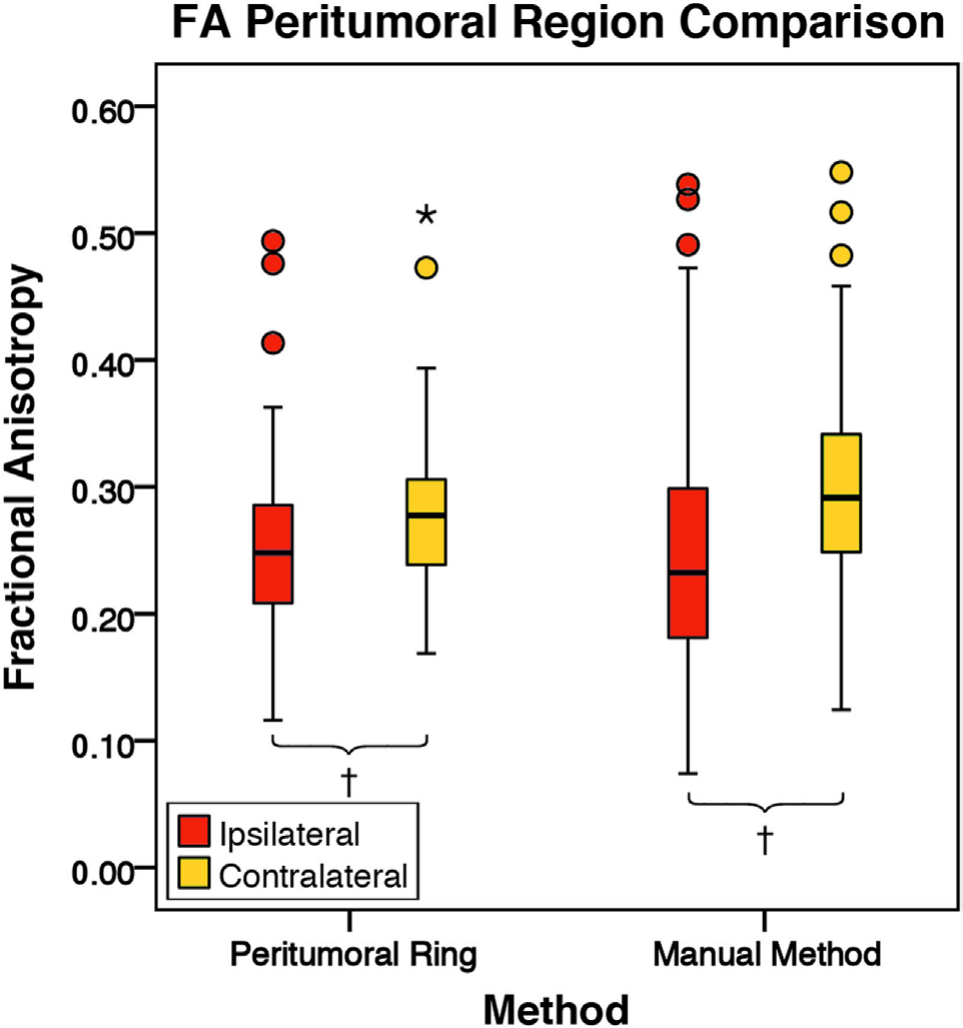
**The mean fractional anisotropy (FA) values for the peritumoral regions and their contralateral counterpart (*n* = 84)**. The boxes represent the interquartile range (IQR) with the median denoted as a horizontal line. Data points beyond the whiskers (1.5 × IQR) were considered outliers (circles), and extreme cases (beyond 3 × IQR) were denoted as stars. These data points were not excluded from the statistical analysis. Using the peritumoral ring method, the ipsilateral and contralateral regions of interest (ROIs) had peritumoral mean FA values of 0.25 ± 0.07 and 0.28 ± 0.06 (SD), respectively. Using the manual method, the ipsilateral and contralateral ROIs had peritumoral mean FA values of 0.25 ± 0.10 and 0.30 ± 0.08 (SD), respectively. FA was significantly lower in the ipsilateral peritumoral ROIs than the contralateral ROIs for both methods (*p* = 0.001; ^†^*p* < 0.01).

**Figure 5:**
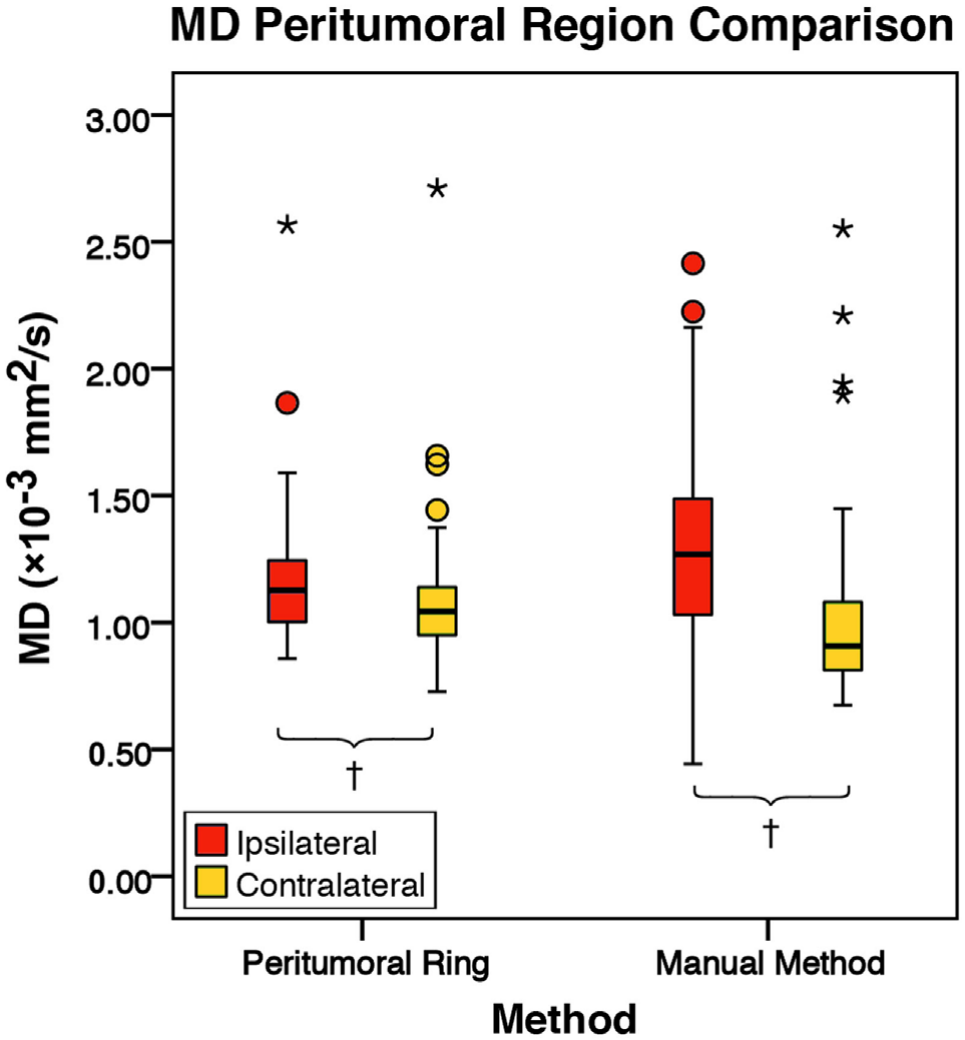
**The mean diffusivity (MD) values for the peritumoral regions and their contralateral counterpart (*n* = 84)**. The boxes represent the interquartile range (IQR) with the median denoted as a horizontal line. Data points beyond the whiskers (1.5 × IQR) were considered outliers (circles), and extreme cases (beyond 3 × IQR) were denoted as stars. These data points were not excluded from the statistical analysis. Using the peritumoral ring method, the ipsilateral and contralateral regions of interest (ROIs) had peritumoral mean MD values of 1.16 ± 0.24 and 1.08 ± 0.24 × 10^−3^ mm^2^/s (SD), respectively. Using the manual method, the ipsilateral and contralateral ROIs had peritumoral mean MD values of 1.29 ± 0.35 and 1.01 ± 0.32 × 10^−3^ mm^2^/s (SD), respectively. MD was significantly higher in the ipsilateral peritumoral ROIs than the contralateral ROIs for both the manual sample and peritumoral ring method (*p* = 0.001; *p* = 0.050; ^†^*p* < 0.01).

**Figure 6:**
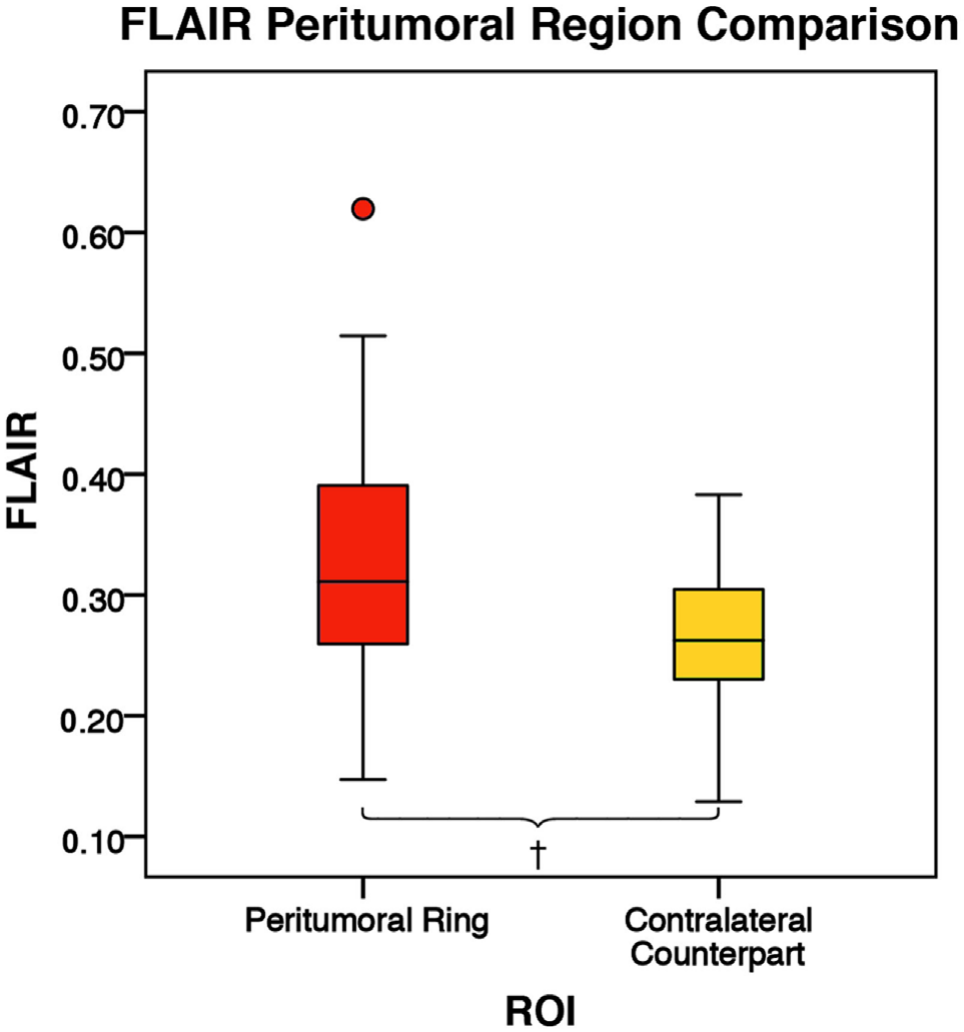
**The mean fluid-attenuated inversion recovery (FLAIR) values for the peritumoral regions and their contralateral counterpart (*n* = 84)**. The boxes represent the interquartile range (IQR) with the median denoted as a horizontal line. Data points beyond the whiskers (1.5 × IQR) were considered outliers (circles) and were not excluded from the statistical analysis. Using the peritumoral ring method, the ipsilateral and contralateral regions of interest (ROIs) had peritumoral mean FLAIR values of 0.33 ± 0.09 and 0.26 ± 0.06 (SD), respectively. FLAIR was significantly higher in the ipsilateral peritumoral ring than the contralateral peritumoral ring (*p* = 0.001; ^†^*p* < 0.01).

**Figure 7:**
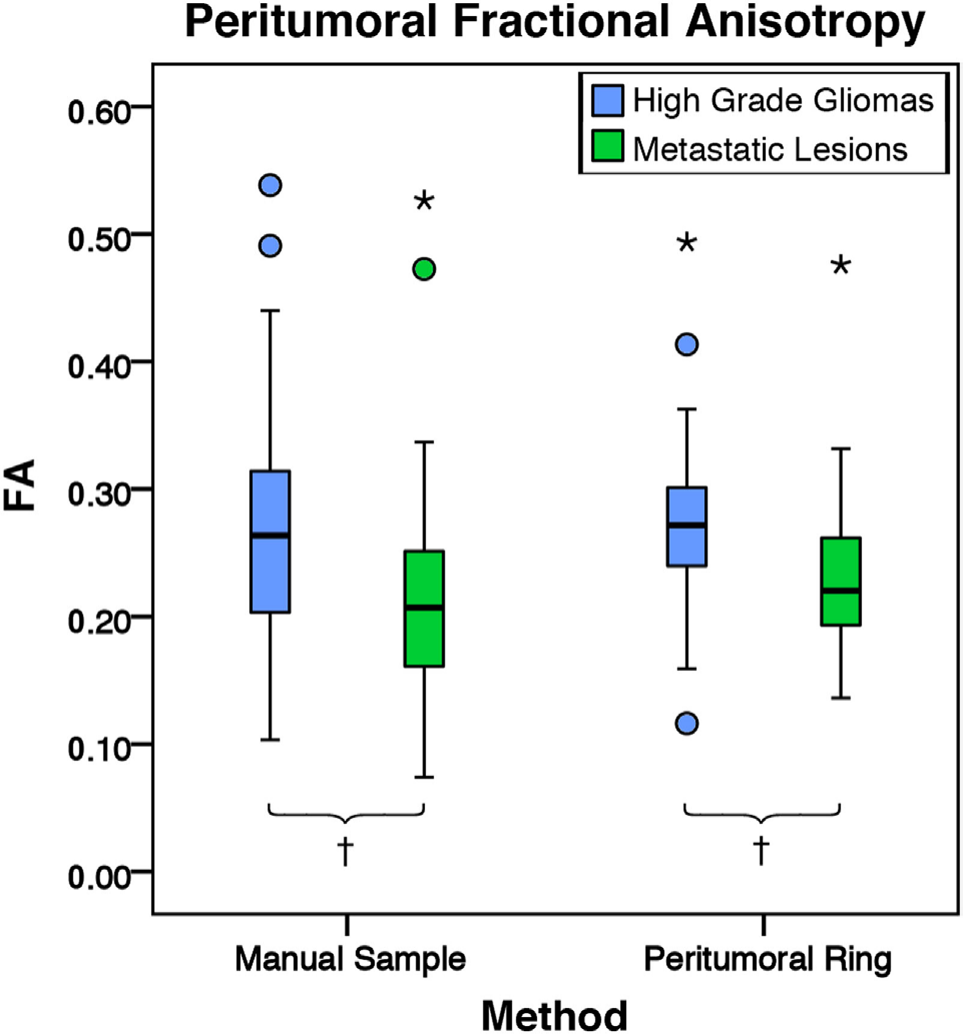
**The mean fractional anisotropy (FA) values for high-grade gliomas (*n* = 40) and metastatic lesions (*n* = 44)**. The boxes represent the interquartile range (IQR) with the median denoted as a horizontal line. Data points beyond the whiskers (1.5 × IQR) were considered outliers (circles), and extreme cases (beyond 3 × IQR) were denoted as stars. These data points were not excluded from the statistical analysis. For the manual sample method, the high-grade gliomas and metastatic lesions had peritumoral mean FA values of 0.27 ± 0.10 and 0.22 ± 0.09 (SD), respectively. The high-grade gliomas were found to have a significantly higher peritumoral FA mean difference of 0.05 95% CI (0.01, 0.09) than metastases (*p* = 0.009). For the peritumoral ring method, the high-grade gliomas and metastatic lesions had mean peritumoral FA values of 0.32 ± 0.09 and 0.29 ± 0.09 (SD), respectively. The high-grade gliomas were found to have a significantly higher peritumoral FA mean difference of 0.04 95% CI (0.01, 0.08) than metastases (*p* = 0.004; ^†^*p* < 0.01).

**Figure 8:**
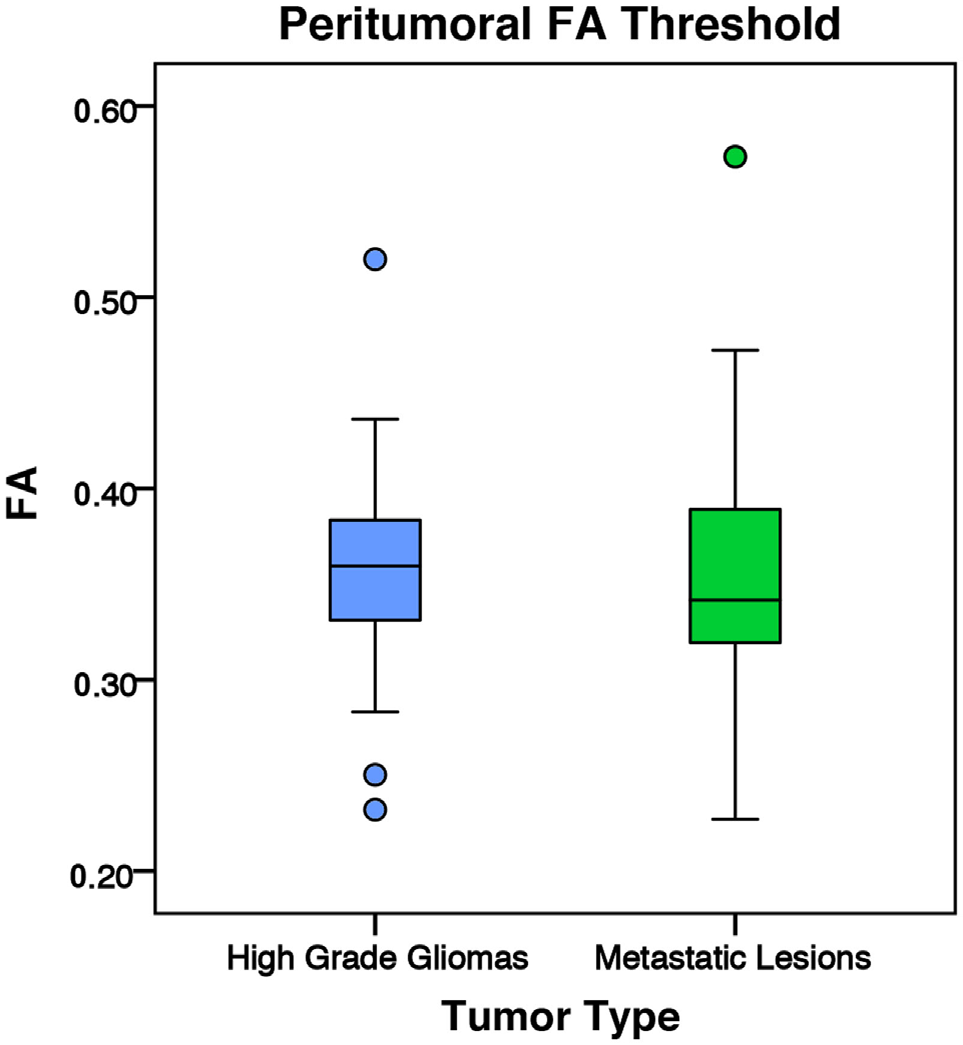
**The mean fractional anisotropy (FA) values for high-grade gliomas (*n* = 40) and metastatic lesions (*n* = 44) after eliminating voxels below 0.2**. The boxes represent the interquartile range (IQR) with the median denoted as a horizontal line. Data points beyond the whiskers (1.5 × IQR) were considered outliers (circles) and were not excluded from the statistical analysis. For the manual sample method, the high-grade gliomas and metastatic lesions had peritumoral mean FA values of 0.36 ± 0.05 and 0.35 ± 0.06 (SD), respectively. There was no significant difference between the tumor types (*p* = 0.799).

**Figure 9:**
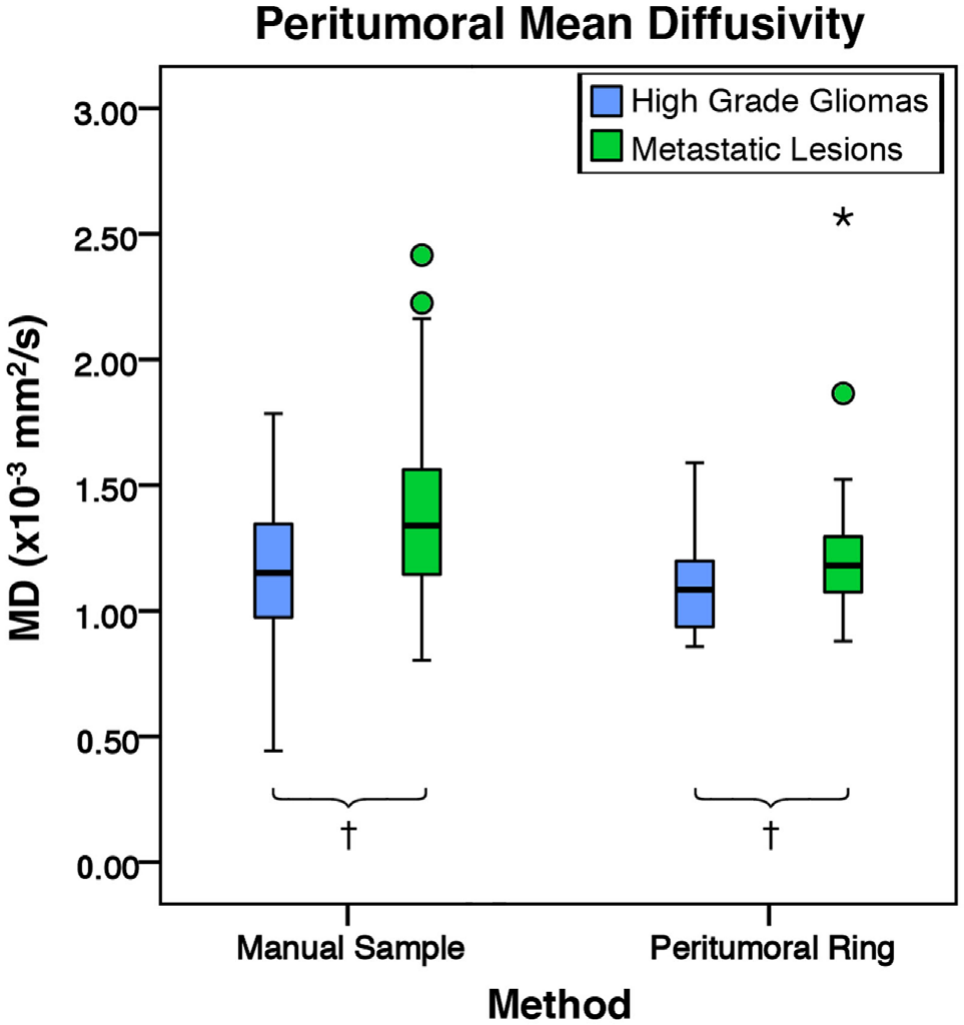
**The mean diffusivity (MD) values for high-grade gliomas (*n* = 40) and metastatic lesions (*n* = 44)**. The boxes represent the interquartile range (IQR) with the median denoted as a horizontal line. Data points beyond the whiskers (1.5 × IQR) were considered outliers (circles), and extreme cases (beyond 3 × IQR) were denoted as stars. These data points were not excluded from the statistical analysis. For the manual method, the high-grade gliomas and metastatic lesions had peritumoral mean MD values of 1.17 ± 0.27 and 1.40 ± 0.38 × 10^−3^ mm^2^/s (SD), respectively. The metastases were found to have a significantly higher peritumoral MD mean difference of 0.23 × 10^−3^ mm^2^/s 95% CI (0.08, 0.37) than high-grade gliomas (*p* = 0.002). For the peritumoral ring method, the high-grade gliomas and metastatic lesions had mean peritumoral MD values of 1.08 ± 0.17 and 1.22 ± 0.27 × 10^−3^ mm^2^/s (SD), respectively. The metastases were found to have a significantly higher peritumoral MD mean difference of 0.14 × 10^−3^ mm^2^/s 95% CI (0.04, 0.24) than high-grade gliomas (*p* = 0.007; ^†^*p* < 0.01).

**Figure 10:**
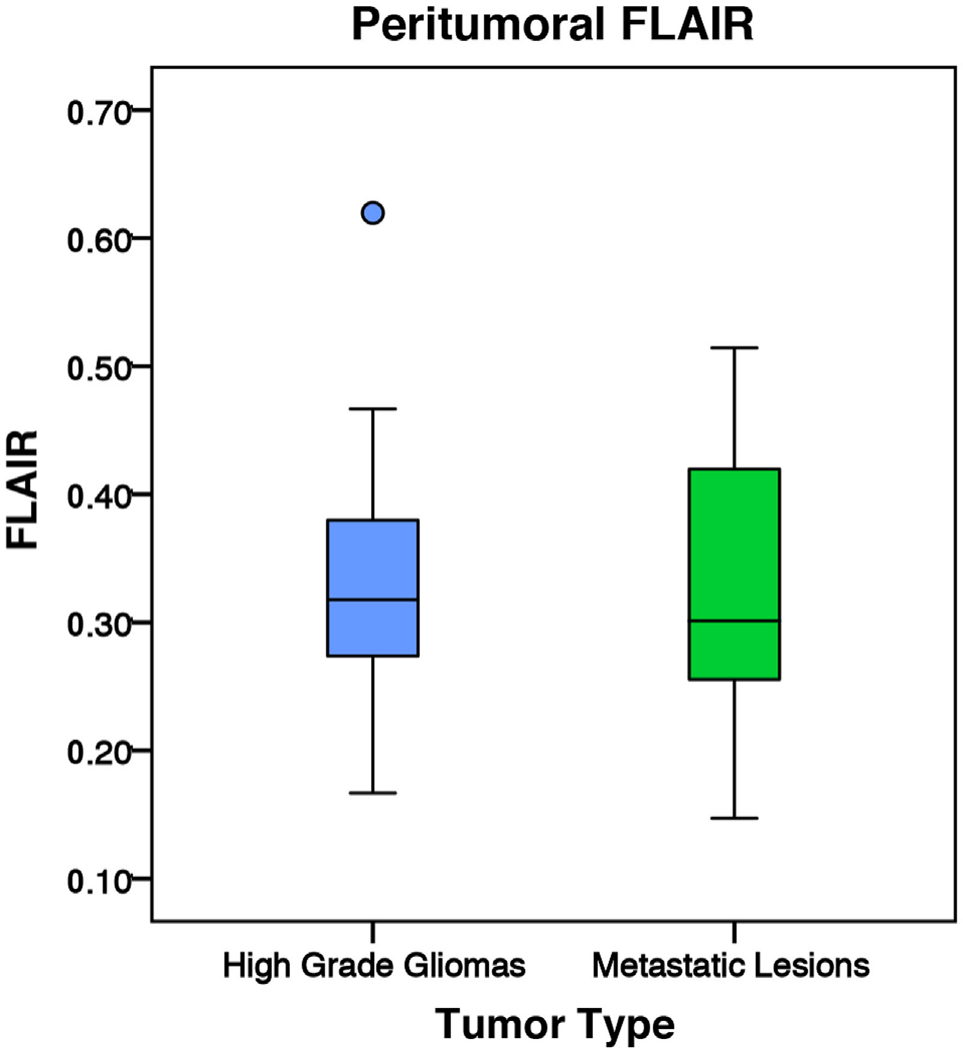
**The mean fluid-attenuated inversion recovery (FLAIR) values for high-grade gliomas (*n* = 35) and metastatic lesions (*n* = 30)**. The boxes represent the interquartile range (IQR) with the median denoted as a horizontal line. Data points beyond the whiskers (1.5 × IQR) were considered outliers (circles) and were not excluded from the statistical analysis. Using the peritumoral ring method, the high-grade gliomas and metastatic lesions had normalized peritumoral mean FLAIR values of 1.23 ± 0.22 and 1.25 ± 0.30 (SD), respectively. There was no significant difference in peritumoral FLAIR between the two tumor types (*p* = 0.764).

There was no significant difference in mean intratumoral FA, MD, or FLAIR between high-grade gliomas and metastases (Figures S1–S3 in Supplementary Material). For both the manual sample and peritumoral ring method, the ipsilateral peritumoral ROIs had significantly higher MD and significant lower FA than their contralateral counterparts (Figures 4 and 5). The peritumoral ring method showed that FLAIR intensity in the ipsilateral ROIs was significantly greater than their contralateral counterparts (Figure 6). The high-grade gliomas had significantly higher peritumoral FA than the metastases for both the manual sample and peritumoral ring method (Figure 7). However, when restricting analysis to voxels with a FA greater than 0.2 using the peritumoral ring method, there was no significant difference in peritumoral FA between the tumor types (Figure 8). Both methods showed that the peritumoral MD was significantly lower in high-grade gliomas than in metastases (Figure 9). The peritumoral ring method did not detect any significant peritumoral FLAIR difference between tumor types (Figure 10).

### ROC Analysis

Table 2 shows the optimal FA and MD thresholds to distinguish between tumor types for each of the predictive models for both the manual sample method and peritumoral ring method. When utilizing only the FA threshold for the predictive model, both the manual sample method and peritumoral ring method had an optimal threshold of 0.24 that provided the maximum AUC of 70.6 and 71.5%, respectively. The sensitivity, specificity, and accuracy were 68.2, 72.9, and 70.7% for the manual method and 65.9, 77.1, and 71.7% for the peritumoral ring method, respectively. For the MD threshold only predictive model, the optimal MD threshold was to be 0.0013 and 0.0010 mm^2^/s for the manual sample method and peritumoral ring method. The optimal MD threshold provided sensitivity, specificity, accuracy, and AUC of 56.8, 72.9, 65.2, and 65.2% for the manual method and 88.6, 39.6, 63.0, and 64.1% for the peritumoral ring method, respectively. When applying both the MD and FA thresholds in conjunction, the most optimal MD and FA threshold was found to be 0.0001 mm^2^/s and 0.24. The optimal MD and FA thresholds provided sensitivity, specificity, accuracy, and AUC of 68.2, 72.9, 70.7, and 70.6% for the manual method and 65.9, 77.1, 71.7, and 71.5% for the peritumoral ring method, respectively. The optimal MD and FA thresholds were verified by a ROC curves (Figures S4–S7 in Supplementary Material).

**Table 2:** **Optimal threshold results for predictive models**.

	FA CV	MD CV (mm^2^/s)	Sensitivity (%)	Specificity (%)	Accuracy (%)	AUC (%)
**Manual sampling method**						
FA threshold	0.24	–	68.2	72.9	70.7	70.6
MD threshold	–	0.0013	56.8	72.9	65.2	65.2
Both thresholds	0.24	0.0001	68.2	72.9	70.7	70.6
**Peritumoral ring method**						
FA threshold	0.24	–	65.9	77.1	71.7	71.5
MD threshold	–	0.0010	88.6	39.6	63.0	64.1
Both thresholds	0.24	0.0001	65.9	77.1	71.7	71.5

*FA, fractional anisotropy; MD, mean diffusivity; CV, cutoff value; AUC, area under the curve*.

The FA values were shown to be able to differentiate the tumor types more effectively than MD or FLAIR. For both methods, MD was able to slightly differentiate between the tumor types. Including an MD threshold in addition to the FA threshold in the predictive model did not increase the reliability of the predictive model.

## Discussion

In this study, we explored the efficacy of using DTI to differentiate high-grade glioma and intracranial metastasis. It is desirable to be able to reliably differentiate these two types of lesions non-invasively. For example, for patients with medical comorbidities that increase the risk for perioperative complications, surgical resection can be avoided if a diagnosis can be achieved non-invasively. In some cases, the lesion is small and deeply seated, and it may be challenging to obtain diagnostic specimen with surgery. Finally, even in the case where surgical resection is planned, the knowledge of the tumor type may help the surgeon with surgical planning and intraoperative decision-making since these two types of tumors have different interactions with surrounding brain tissue.

In our study, we did not find any significant difference in mean intratumoral FA, MD, or FLAIR between high-grade gliomas and metastases. These results are supported by the existing literature (4, 6, 7). We did find, however, with both the manual sample method and peritumoral ring method, that high-grade gliomas had a significantly higher peritumoral FA and significantly lower MD than metastases. We believe differences in how the two types of tumors interact with surrounding tissues have led to these differences in DTI values.

Gliomas and metastatic lesions are both known to cause vasogenic edema in the surrounding tissue (1), which is hyperintense in T2 and FLAIR MR images. We neither found any significant difference in FLAIR intensity intratumorally nor peritumorally between the high-grade gliomas and metastases. Bodsch et al. found through biopsy that water content was almost identical in intratumoral and peritumoral tissues between glioblastomas and metastases (36). They examined 39 tumor samples (30 glioblastomas and 9 metastases) and 20 edema samples (16 glioblastomas and 4 metastases). These results, including ours, pointed to no differences in amount of surrounding edema between the tumor types. In one study, Tang et al. found tumors with abnormal FLAIR intensity surrounding the non-enhancing portion of the tumor were more likely to indicate a glioma than a metastasis (2). This particular finding was only limited to 19 subjects (16 gliomas and 3 metastases) out of 70 enrolled subjects.

In our study, we found the vasogenic edema altered the values of FA and MD. We found both high-grade gliomas and metastatic lesions exhibited higher peritumoral MD and lower peritumoral FA compared to their contralateral normal counterparts. An increase in extracellular water content surrounding the tumors is the likely mechanism responsible for a higher MD and lower FA within peritumoral regions when compared to normal tissue (1, 10, 12). These results including ours seemed to suggest that the peritumoral edema had opposite effect on FA and MD values. Lu et al. hypothesized that peritumoral FA and MD values are inversely related by their dependence on free extracellular water content. Among meningioma and metastatic patients, the authors showed an inverse linear relationship between FA and MD (7).

Our results showed significantly more pronounced vasogenic edema effects on the FA and MD in metastases than in gliomas with high-grade gliomas demonstrating a significantly higher peritumoral FA and significantly lower MD than metastases. Lu et al. hypothesized that peritumoral FA in gliomas was also affected by tumor infiltration, which did not occur in metastases or meningiomas. They suggested that infiltrative tumor cells may disrupt white matter tracts causing a decrease in anisotropic diffusion (10). Despite this hypothesis, the authors did not find significant difference in peritumoral FA values among gliomas, meningiomas, and metastases. The limitations provided by Lu et al. of small population size and subjective ROI placement along white matter tracts could potentially explain our disparate findings (10). Our results suggested that the presence of tumor infiltration exhibited by gliomas led to a higher peritumoral FA and lower peritumoral MD than metastases. The presence of tumor cells within the increased extracellular water content may have led to an increase in anisotropic diffusion and a decrease in MD.

Another possible explanation for the difference could have been that the significantly larger high-grade gliomas may have pushed and compacted surrounding white matter tracts. It is possible that a larger tumor may increase the anisotropy of its surrounding tissue. In our study, high-grade gliomas had significantly higher tumor-to-brain ratio than metastases (Table 1). Interestingly, when targeting the white matter by applying a conventional 0.2 FA cutoff value, there was no significant difference detected between high-grade gliomas and metastases (Figure 8). This would suggest the peritumoral tissue may have been altered significantly from the combination of the tumor mass effect, vasogenic edema, and tumor infiltration. In this case, utilizing conventional DTI values to differentiate between the gray and white matter may no longer be applicable. Although the vasogenic edema seems to affect both FA and MD, the tumor infiltration present in the high-grade gliomas appears to be the differential mechanism that leads to a higher peritumoral FA and lower peritumoral MD.

Using the ROC analysis, we found that a peritumoral FA threshold is better than a peritumoral MD threshold at differentiating the two tumor types. Combining the two metrics did not improve the performance compared to using the FA threshold alone. Obviously, the thresholds of FA and MD found in this study are only pertinent to this particular DTI dataset. Another DTI dataset acquired with a different imaging protocol or at another institution will likely require the same imaging processing protocol outlined in Section “Materials and Methods” to establish its relevant FA and MD threshold for differentiating the two tumor types. Furthermore, neither threshold provided perfect sensitivity and specificity. The final determination of the tumor type cannot be based on these thresholds alone.

To the best of our knowledge, this is the first comparison study between manual sample method and a peritumoral ring method. Our novel semiautomated peritumoral ring method circumvents the need for an expert to hand draw ROIs surrounding the tumor. In addition, it provides a more objective ROI selection that is larger and more inclusive. Our study suggested the effect on peritumoral FA and MD values from tumor might not have been limited to the white matter tracts. In contrast using a hand-drawn peritumoral ring method, Papageorgiou et al. found gliomas had higher FA than metastases when including the entire peritumoral region (11). As suggested by other groups (8, 21, 37), a larger and more inclusive ROI is perhaps more advantageous.

## Conclusion

A novel semiautomated peritumoral ring method was compared to a manual sample method in obtaining DTI metrics to differentiate high-grade gliomas and metastatic lesions. Both methods were able to demonstrate a significant difference both with FA and MD metrics between high-grade gliomas and metastases. In our study, FA provided a more sensitive measure in differentiating the tumors than MD. The semiautomated peritumoral ring method allows for a larger more inclusive ROI without a dependence on expertise to hand draw ROIs.

## Ethics Statement

A retrospective cohort study on patients that underwent surgical resection for gliomas and metastases at Louisiana State University Health Sciences Center in Shreveport, LA, USA, between January 2011 and June 2016 was performed following approval from the Institutional Review Board for the Protection of Human Research Subjects.

## Author Contributions

BB, RB, KH, CL, DM, AN, and EG-T: data acquisition; BB, RB, KH, and DM: image processing; BB, KH, PK, and DM: data analysis; BB, KH, PK, and HS: wrote the paper.

## Conflict of Interest Statement

The authors declare that the research was conducted in the absence of any commercial or financial relationships that could be construed as a potential conflict of interest. The handling editor declared a past supervisory role with one of the authors and states that the process nevertheless met the standards of a fair and objective review.
