# Supplementary material for: High-grade Gliomas Exhibit Higher Peritumoral Fractional Anisotropy and Lower Mean Diffusivity than Intracranial Metastases
Source: Front Surg. 2017 Apr 10;4:18. doi: 10.3389/fsurg.2017.00018 (PMC5385351; doi:10.3389/fsurg.2017.00018)
Supplement: Supplementary file 1 [file data_sheet_1.pdf]

SUPPLEMENTARY MATERIAL

High Grade Gliomas Exhibit Higher Peritumoral Fractional Anisotropy and Lower Mean Diffusivity than Intracranial Metastases

Kevin S. Holly, Ben Barker, Derrick Murcia, Rebekah Bennett, Piyush Kalakoti, Christina Ledbetter, Eduardo Gonzalez-Toledo, Anil Nanda, Hai Sun*

*** Correspondence:** Hai Sun: hsun2@lsuhsc.edu

1 Supplementary Figures

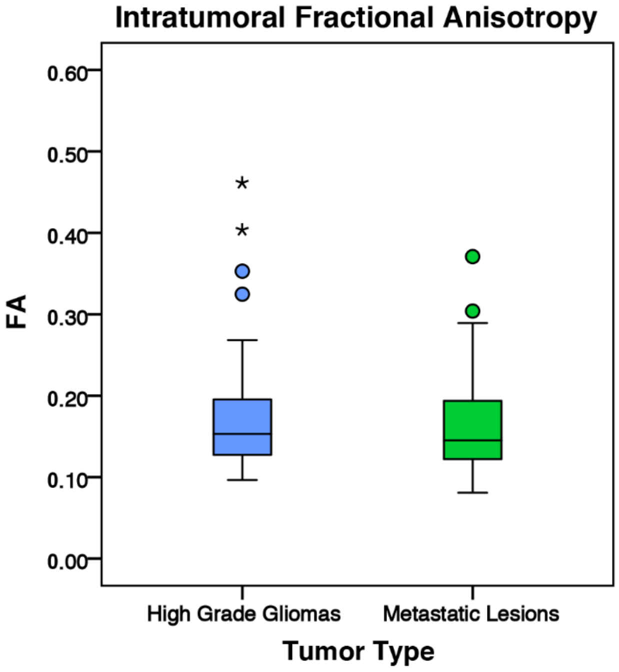

SUPPLEMENTARY FIGURE 1: The mean FA values within the tumor for high grade gliomas (n=40) and metastatic lesions (n=44). The boxes represent the interquartile range (IQR) with the median denoted as a horizontal line. Data points beyond the whiskers (1.5×IQR) were considered outliers (circles) and extreme cases (beyond 3×IQR) were denoted as stars. These data points were not excluded from the statistical analysis. The high grade gliomas and metastatic lesions had mean intratumoral FA values of 0.18 ± 0.08 and 0.16 ± 0.06 (SD), respectively. There was no significant difference in intratumoral FA between the two tumor types ($p = .254$).

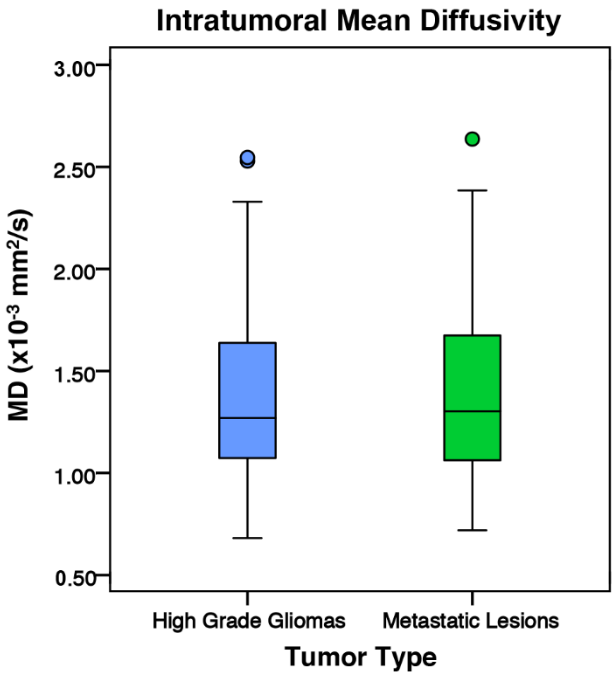

SUPPLEMENTARY FIGURE 2. The mean MD values within the tumor for high grade gliomas (n=40) and metastatic lesions (n=44). The boxes represent the interquartile range (IQR) with the median denoted as a horizontal line. Data points beyond the whiskers (1.5×IQR) were considered outliers (circles) and were not excluded from the statistical analysis. The high grade gliomas and metastatic lesions had mean intratumoral MD values of 1.4 ± 0.03 and 1.39 ± 0.47 $\times 10^{-3}$ mm^2/s (SD), respectively. There was no significant difference in intratumoral MD between the two tumor types ($p = .936$).

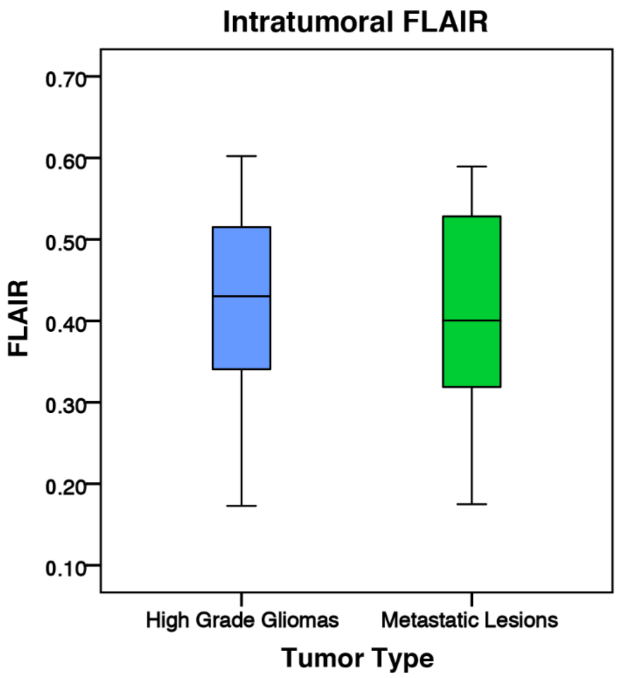

SUPPLEMENTARY FIGURE 3. The mean FLAIR values within for high grade gliomas (n=40) and metastatic lesions (n=44). There were no outliers beyond the whiskers (1.5×IQR). The high grade gliomas and metastatic lesions had normalized mean intratumoral FLAIR values of 1.18 ± 0.28 and 1.07 ± 0.30 (SD), respectively. There was no significant difference in intratumoral FLAIR between the two tumor types ($p = .452$).

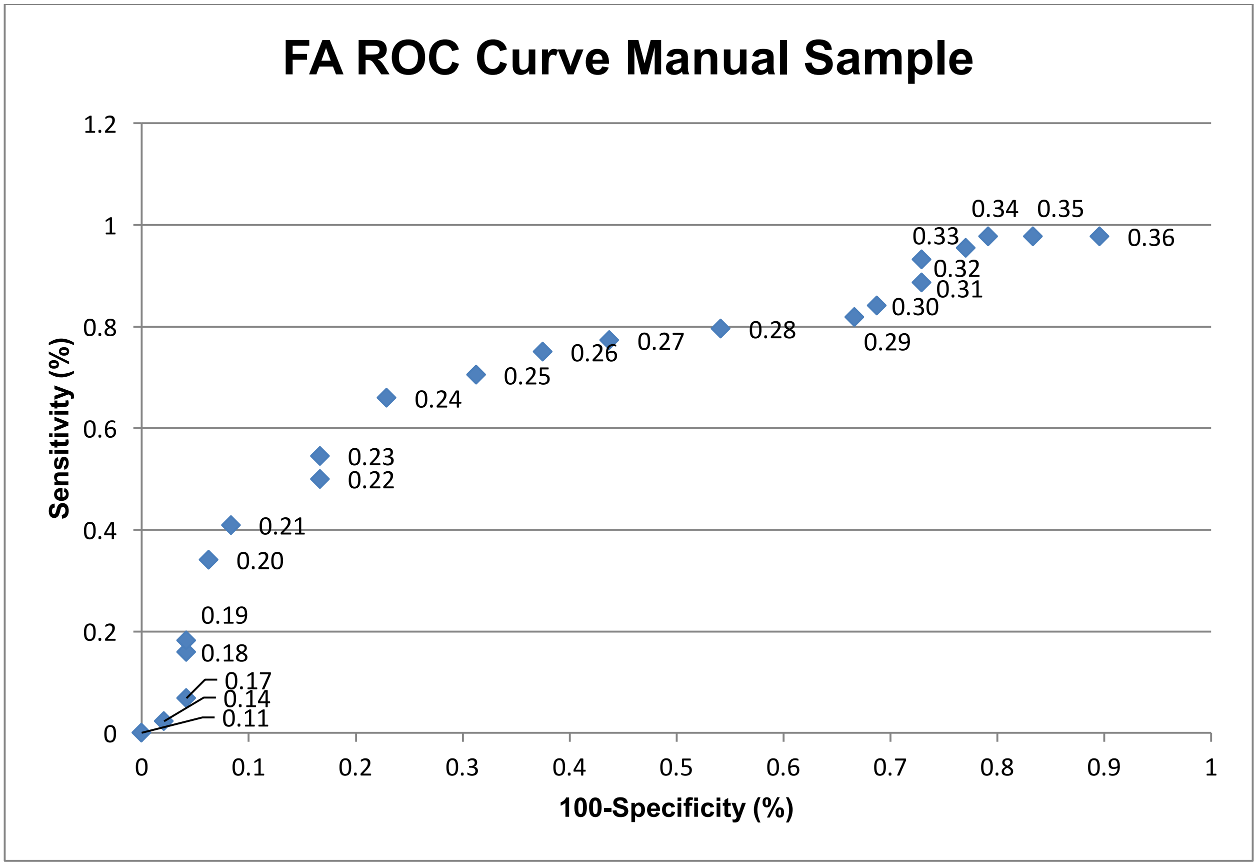

SUPPLEMENTARY FIGURE 4. ROC curve for FA threshold using the manual sample method where FA is the only threshold in the predictive model. The chosen FA threshold value of 0.24 can be found on the top left of the ROC curve indicating a reasonable value.

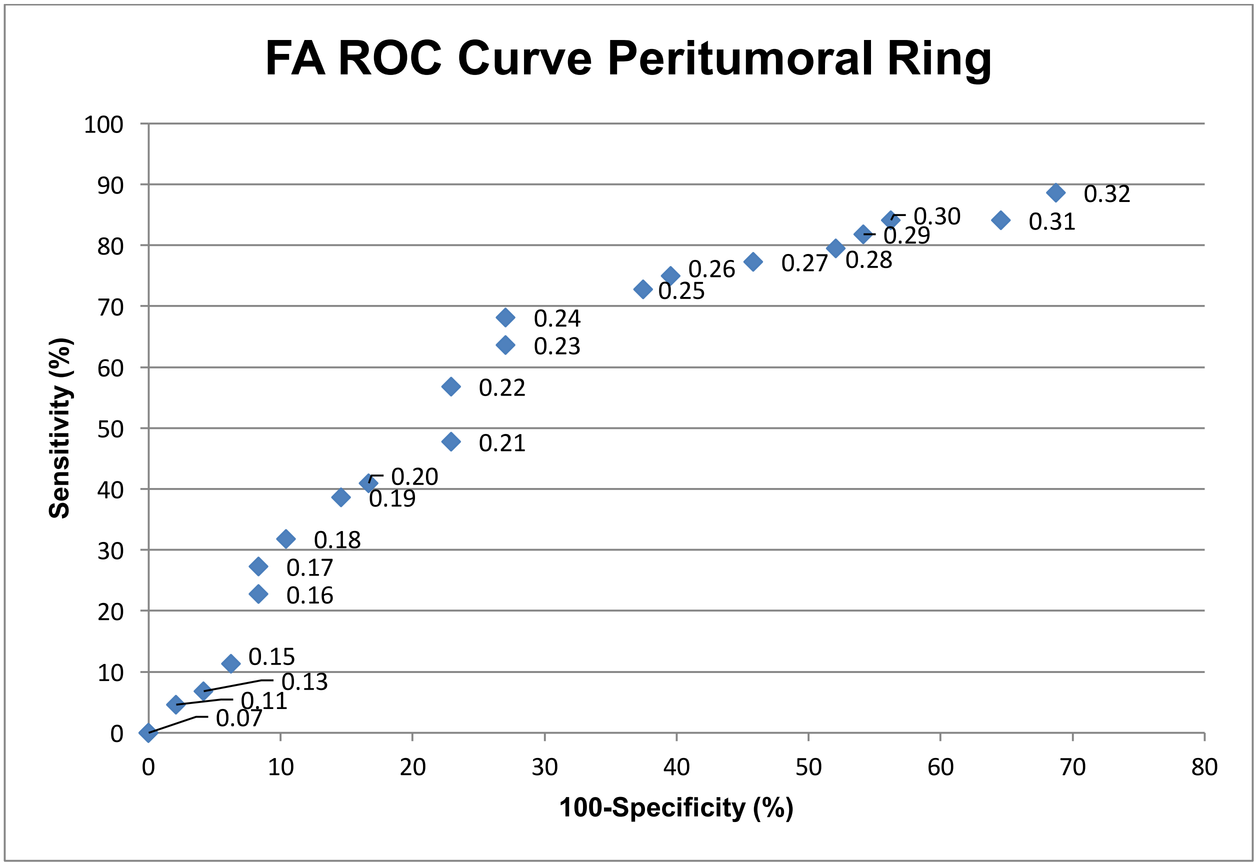

SUPPLEMENTARY FIGURE 5. ROC curve for FA threshold using the peritumoral ring method where FA is the only threshold in the predictive model. The chosen FA threshold value of 0.24 can be found on the top left of the ROC curve indicating a reasonable value.

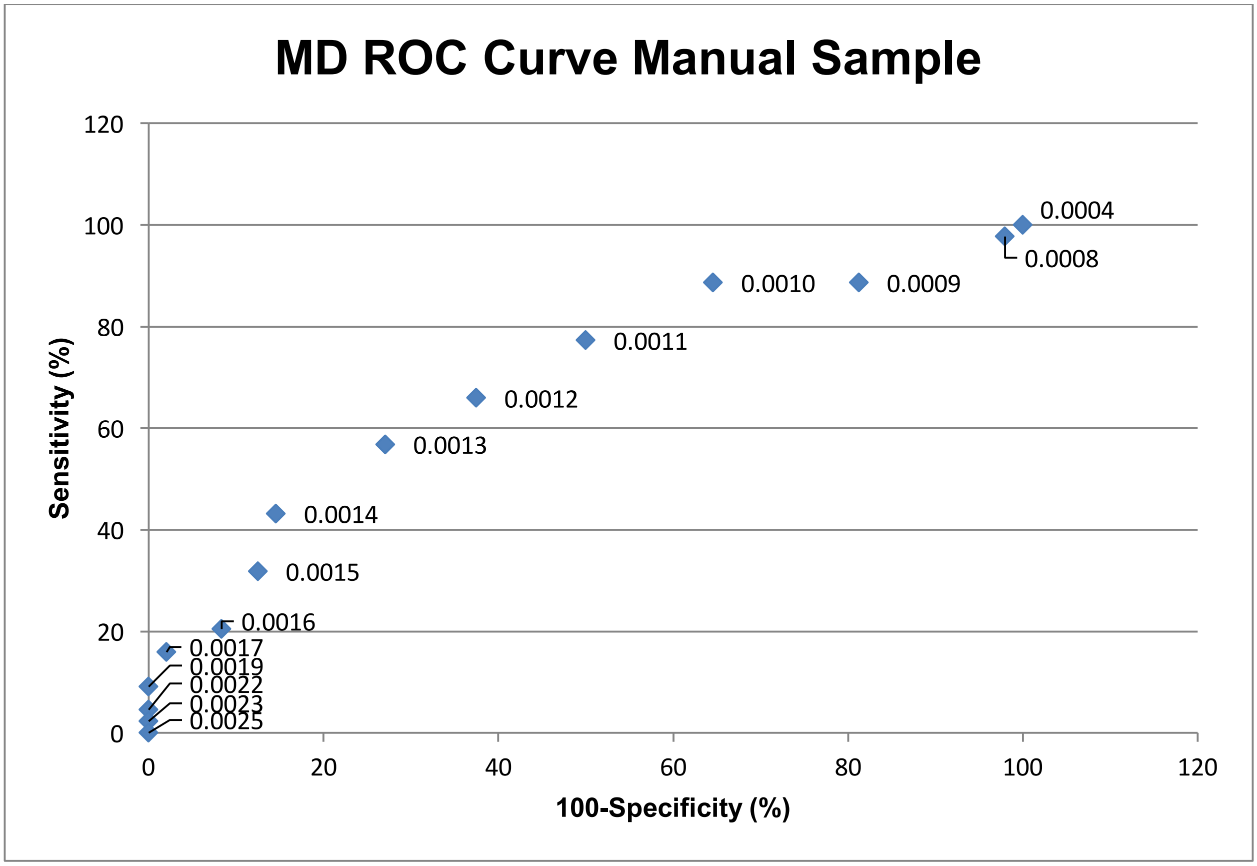

SUPPLEMENTARY FIGURE 6. ROC curve for MD threshold using the manual sample method where MD is the only threshold in the predictive model. The chosen MD threshold value of 0.0013 mm^2/s can be found on the top left of the ROC curve indicating a reasonable value

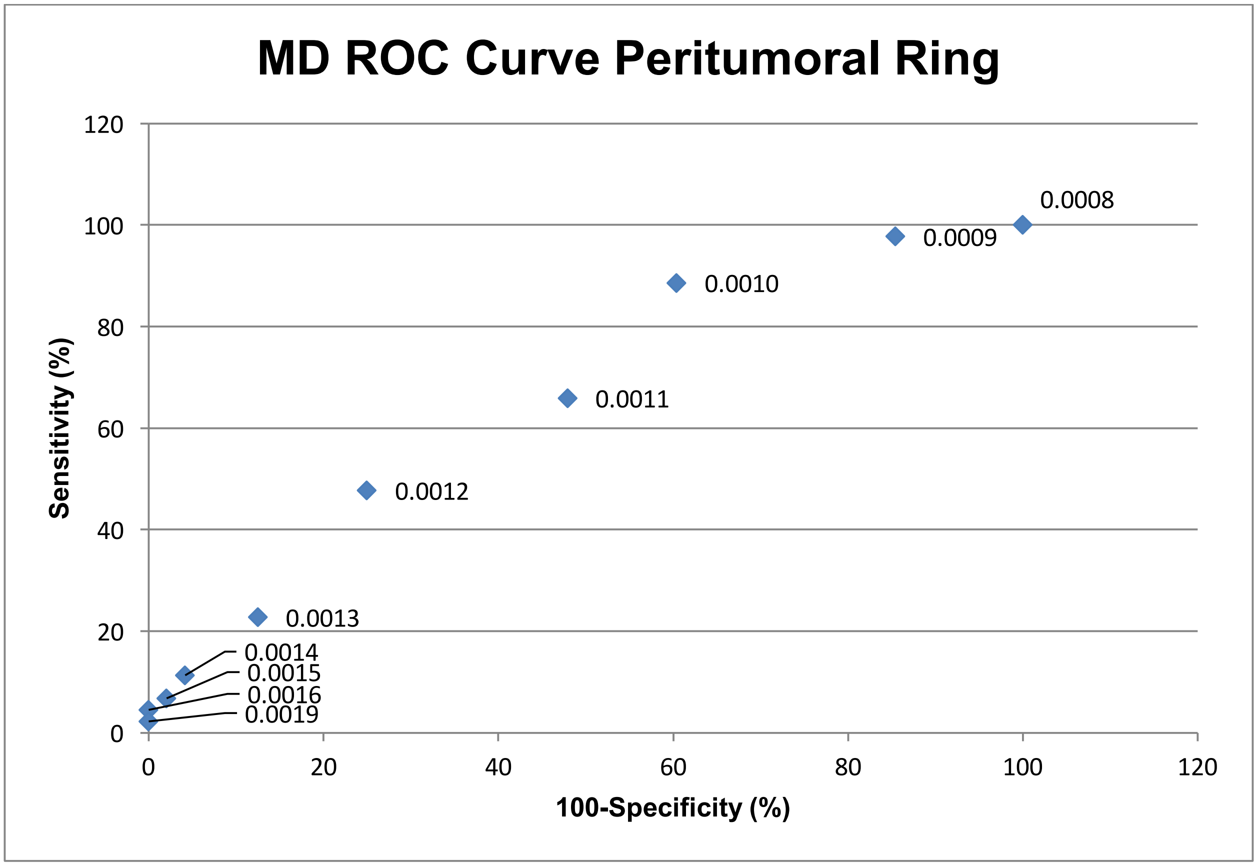

SUPPLEMENTARY FIGURE 7. ROC curve for MD threshold using the peritumoral ring method where MD is the only threshold in the predictive model. The chosen MD threshold value of 0.0010 mm^2/s can be found on the top left of the ROC curve indicating a reasonable value.

2 Supplementary Information

2.1 Image Preprocessing

1. Open 3D Slicer 4.1.1

DICOM to NRRD

2. Click the dropdown box that says “Welcome to Slicer”
    - All Modules>DICOM to NRRD Converter
3. Select input DICOM data for DTI DICOMs
4. Select output Directory of your choosing
5. Enter Output Filename
6. Small Gradient Threshold should be 0.2
7. Click “Apply
8. Select input DICOM data for T1 DICOMs
9. Select output Directory of your choosing
10. Enter Output Filename
11. Small Gradient Threshold should be 0.2
12. Click “Apply

Obtaining Fractional Anisotropy and Trace volumes

13. Open subject folder containing .nrrd files.
14. Drag the DTI, T1, and T1n .nrrd files into the window
    - Click “Okay”
    - Images should appear in dark window
15. Click the dropdown box that now says “DICOM to NRRD Converter”
    - All Modules>DWI to DTI Estimation
16. Click the dropdown box next to “Output DTI Volume” and select “Create new DiffusionTensorVolume”
17. Click the same dropdown box and select “Rename current DiffusionTensorVolume”
18. Type SubjectID_DT and click “OK”
    - Ex: S044_DT
19. Click the dropdown box next to “Output Baseline Volume” and select “Create new Volume”
20. Click the same dropdown box and select “Rename current Volume”
21. Type “Baseline” and click “OK”
22. Select “WLS” as Estimation Parameters
23. Click “Apply”
24. Click the dropdown box that now says “DWI to DTI Estimation”
    - All Modules>Diffusion Tensor Scalar Measurements
25. Click the dropdown box next to “Output Scalar Volume” and select “Create new Scalar Volume”

26. Click the same dropdown box and select “Rename current Volume”
27. Type Type SubjectID FA and click “OK”
    - Ex: S044_FA
28. Make sure “FractionalAnisotropy” is selected under Estimation Parameters
29. Click “Apply”
    - The FA image should appear in the dark window.
30. Click the dropdown box next to “Output Scalar Volume” and select “Create new Scalar Volume”
31. Click the same dropdown box and select “Rename current Volume”
32. Type SubjectID_TR and click “OK”
    - Ex: S044_TR
33. Make sure “Trace” is selected under Estimation Parameters
34. Click “Apply”
    - The Trace image should appear in the dark window.

Registering Images

35. Click the dropdown box that now says “Diffusion Tensor Scalar Measurements”.
    - Select Registration>General Registration (BRAINS)
36. Under Input Images, select the Baseline for Fixed Image Volume.
37. Under Input Images, select T1 for Moving Image Volume.
38. Under Output Settings, select Create new Volume for Output Image Volume.
39. Under Output Settings, select Rename current Volume for Output Image Volume and provide name such as “T1_transformed”.
40. Under Registration Phases, check the Rigid (6 DOF) and Affine (12 DOF) boxes.
41. Under Main Parameters, let Number of Samples = 200000, B-Spline Grid Size = 14, 10, 12, and Max Iterations = 1500.
42. Click “Apply”
43. Repeat steps 37-42 for FLAIR image (Note, FA and Trace should already be aligned to tensor baseline).

Find FA and Trace values at specific ROIs

44. Be sure the FA (or Trace) volume is selected as background (bottom dropdown box in slice viewer).
45. Click the dropdown box that now says “DATA”
    - Select Editor
46. For ‘MasterVolume’ select the FA image (or Trace image)
47. Select ‘GenericAnatomyColors’ if dialog box prompts and click ‘Apply’
48. This will create a FA-label (or TR-label) file

49. Select ‘PaintEffect’ under the ‘Edit Selected Label Map’ heading
50. Set Radius to 3 mm
51. Hover mouse over tac on top left of red slice screen to view images being displayed
52. There should be 3 slots
    - FA-label
    - None
    - FA
53. Change ‘None’ to T1
54. Now click once above (anterior to) the tumor, aligning the edge of the circle just outside the edge of the enhanced region. When clicking, you may need to hold down for a second for 3D Slicer to register the click.
55. Now change the color of brush from ‘1’ to ‘2’ or ‘green’ to ‘yellow’
56. Now click once to the right of the tumor
57. Now change the color of brush from ‘2’ to ‘3’ or ‘yellow’ to ‘dark brown-red’
58. Now click once below (posterior to) the tumor
59. Now change the color of brush from ‘3’ to ‘4’ or ‘dark brown-red’ to ‘light blue’
60. Now click once to the left of the tumor
61. Now change the color of brush from ‘4’ to ‘5’ or ‘light blue’ to ‘red’
62. Now click on the contralateral side complementary to Step 52
63. Now change the color of brush from ‘5’ to ‘6’ or ‘red’ to ‘lighter orange-red’
64. Now click on the contralateral side complementary to Step 54
65. Now change the color of brush from ‘6’ to ‘7’ or ‘lighter orange-red’ to ‘light green’
66. Now click on the contralateral side complementary to Step 56
67. Now change the color of brush from ‘7’ to ‘8’ or ‘light green’ to ‘dark red’
68. Now click on the contralateral side complementary to Step 58
69. Click the dropdown box that now says ‘Editor’
    - All Modules>Label statistics
70. For Grayscale Volume, Select FA
71. For Label Map, Select FA_label
72. Click ‘Apply’
73. For dropdown box near Chart, Select ‘Mean’
74. Click ‘Chart’
75. Verify that bars graphs show 8 ROIs as expected. Ignore anatomy names.
76. Click ‘Save’ and save the spreadsheet.
77. Repeat Steps 46-78 for Trace

2.2 Semi-automated Peritumoral Ring Method

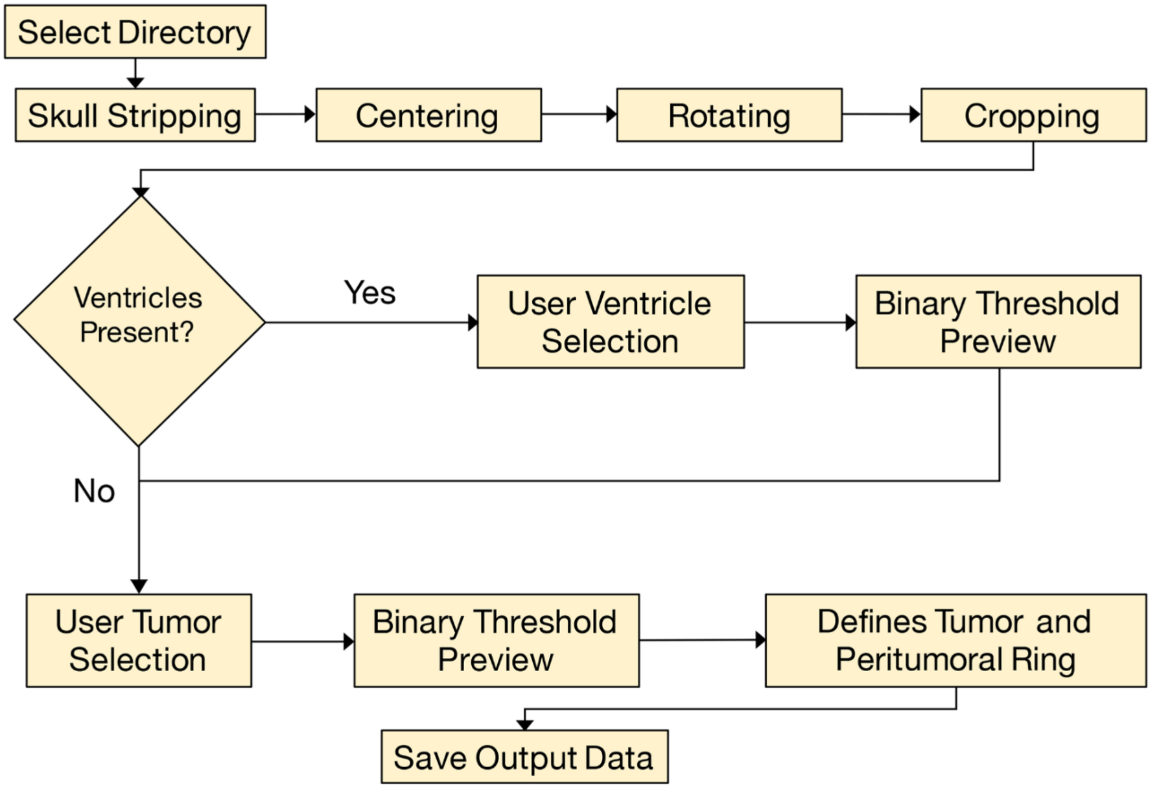

1. Run MATLAB script

2. Following a dialog prompt, select the directory folder containing the folders that hold the NIfTI files for T1, Fraction anisotropy, trace, and FLAIR.

3. After selecting the appropriate slice, the user traces an outline of the brain as shown below and the skull is stripped.

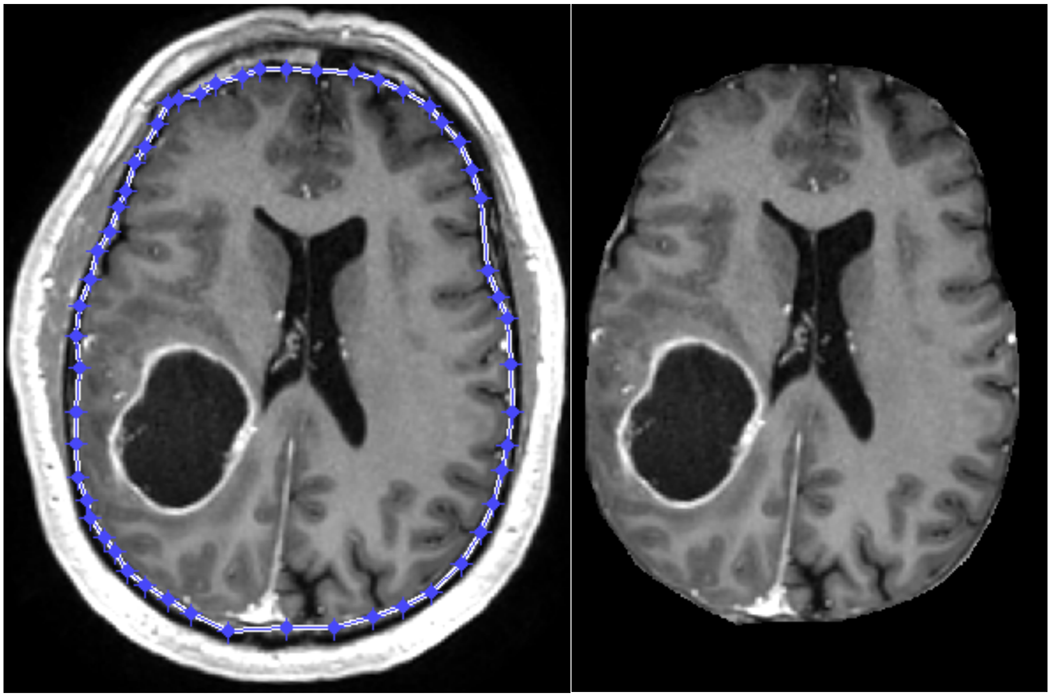

4. Following the dialog prompt, the user selects the midline of the brain and the image is then center, rotated, and cropped.

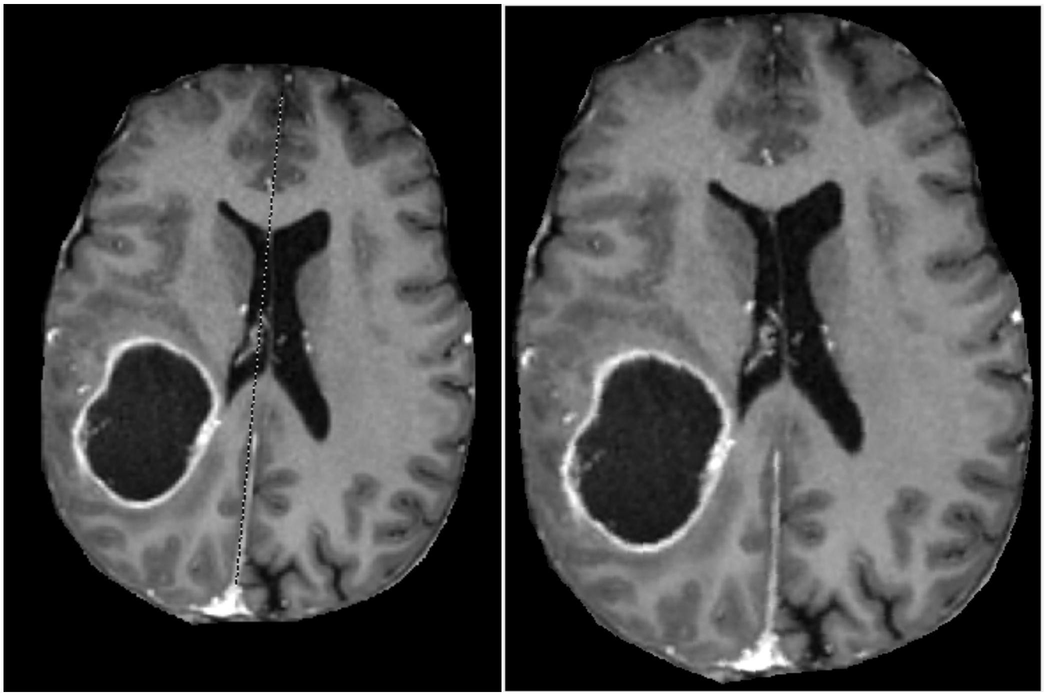

5. If ventricles are present within the slice, the user then roughly selects a region of interest surrounding the ventricles as demonstrated below.

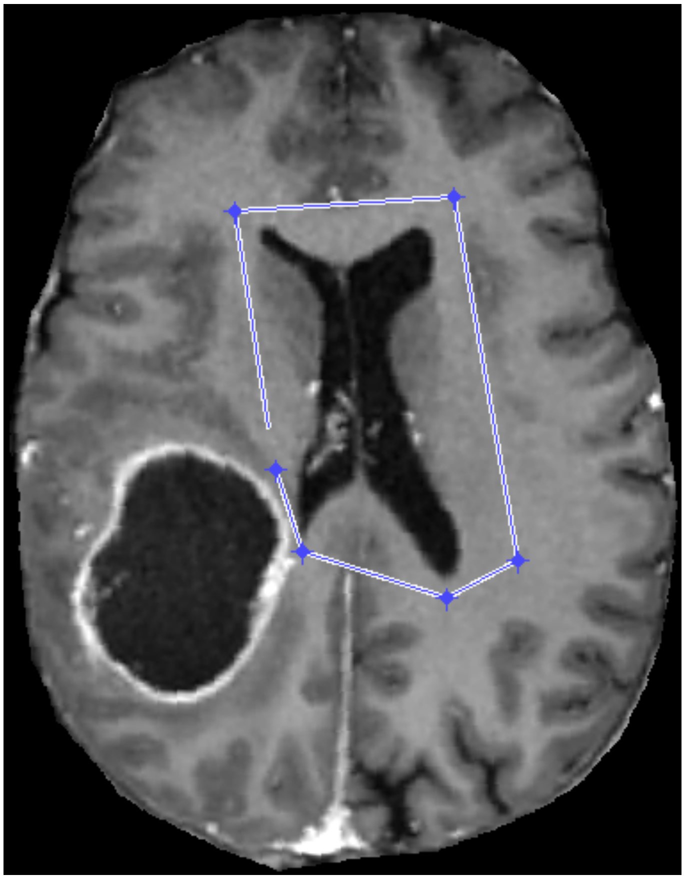

6. A preview screen then pops up for the user to select the appropriate binary threshold.

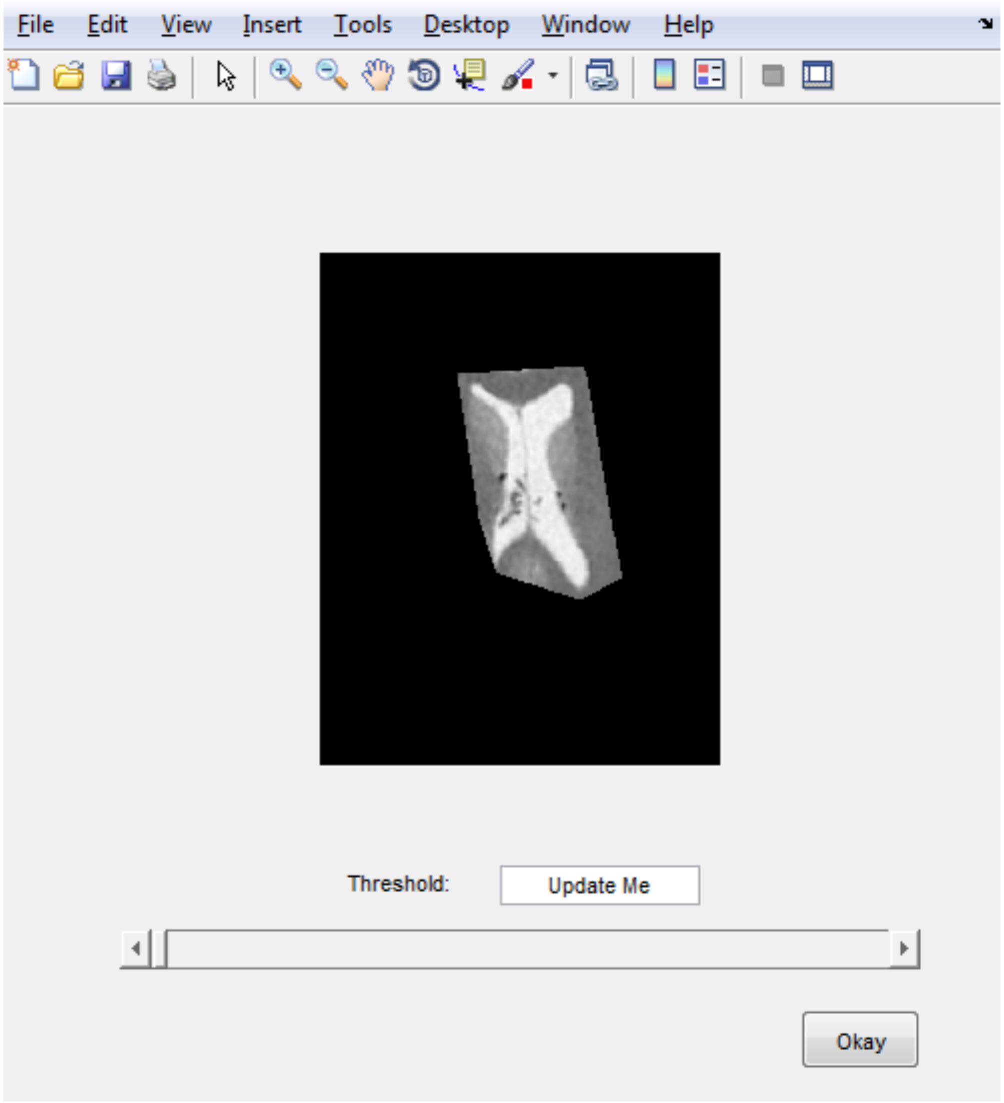

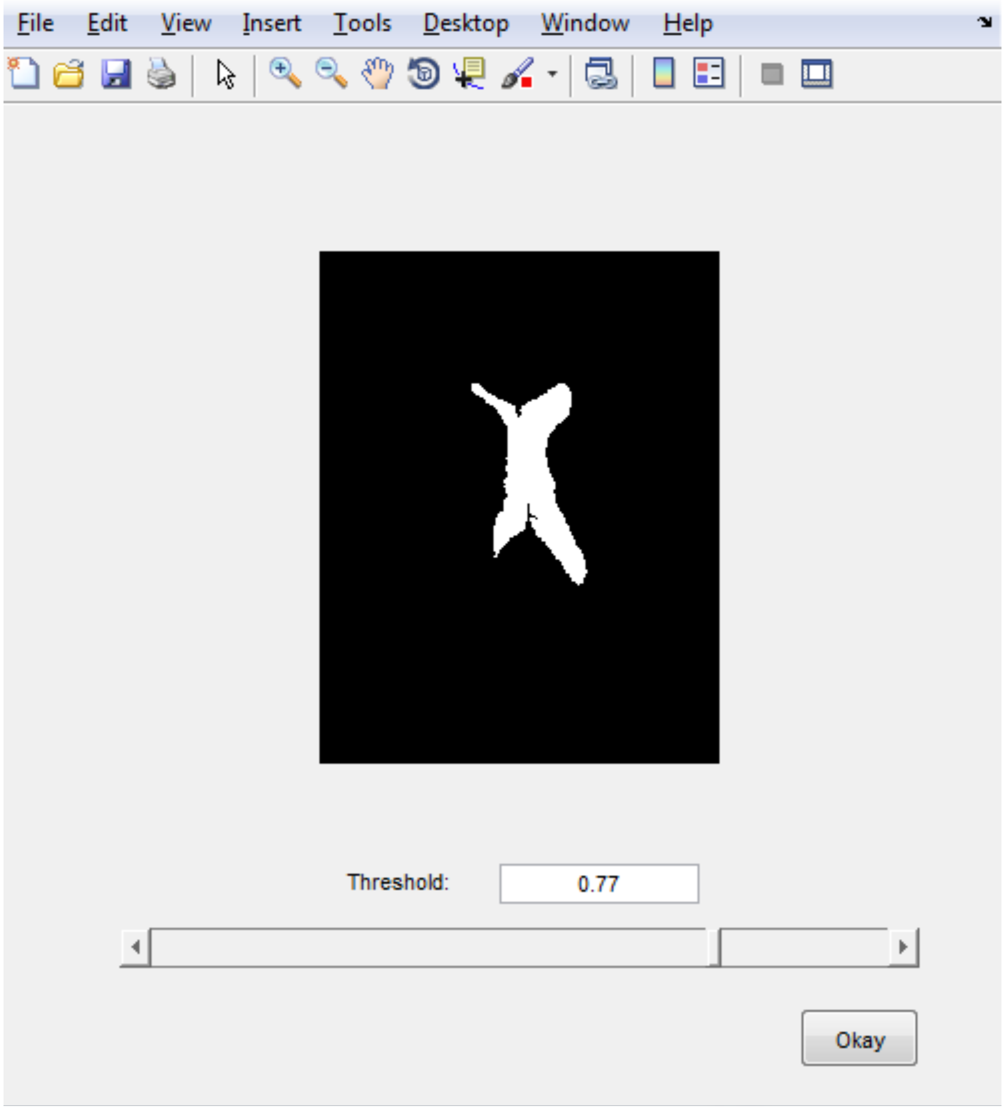
File Edit View Insert Tools Desktop Window Help
Threshold:
0.77
Okay

7. After removing the ventricles from the image, the user selects a ROI surrounding the tumor which is refined by a binary threshold selection.

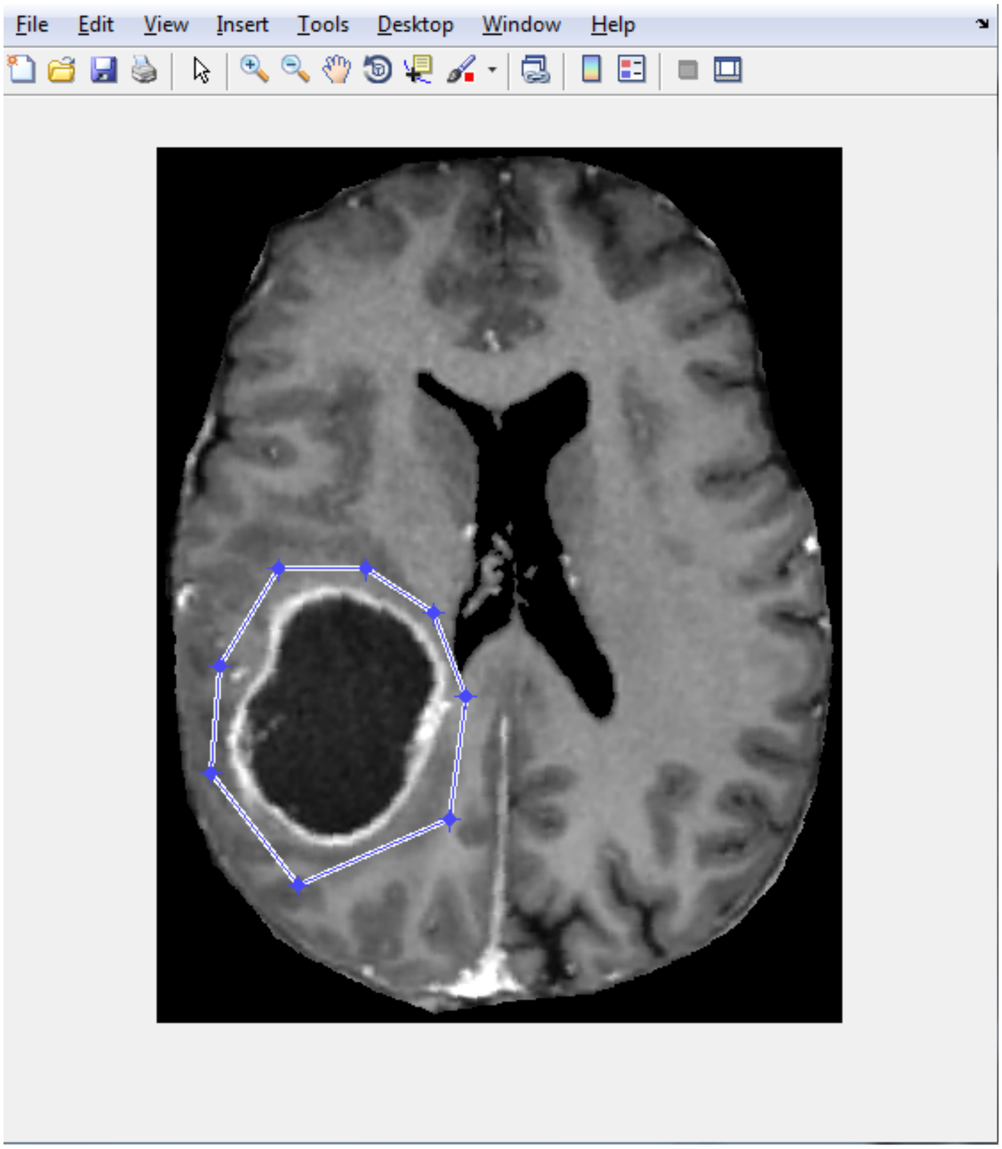

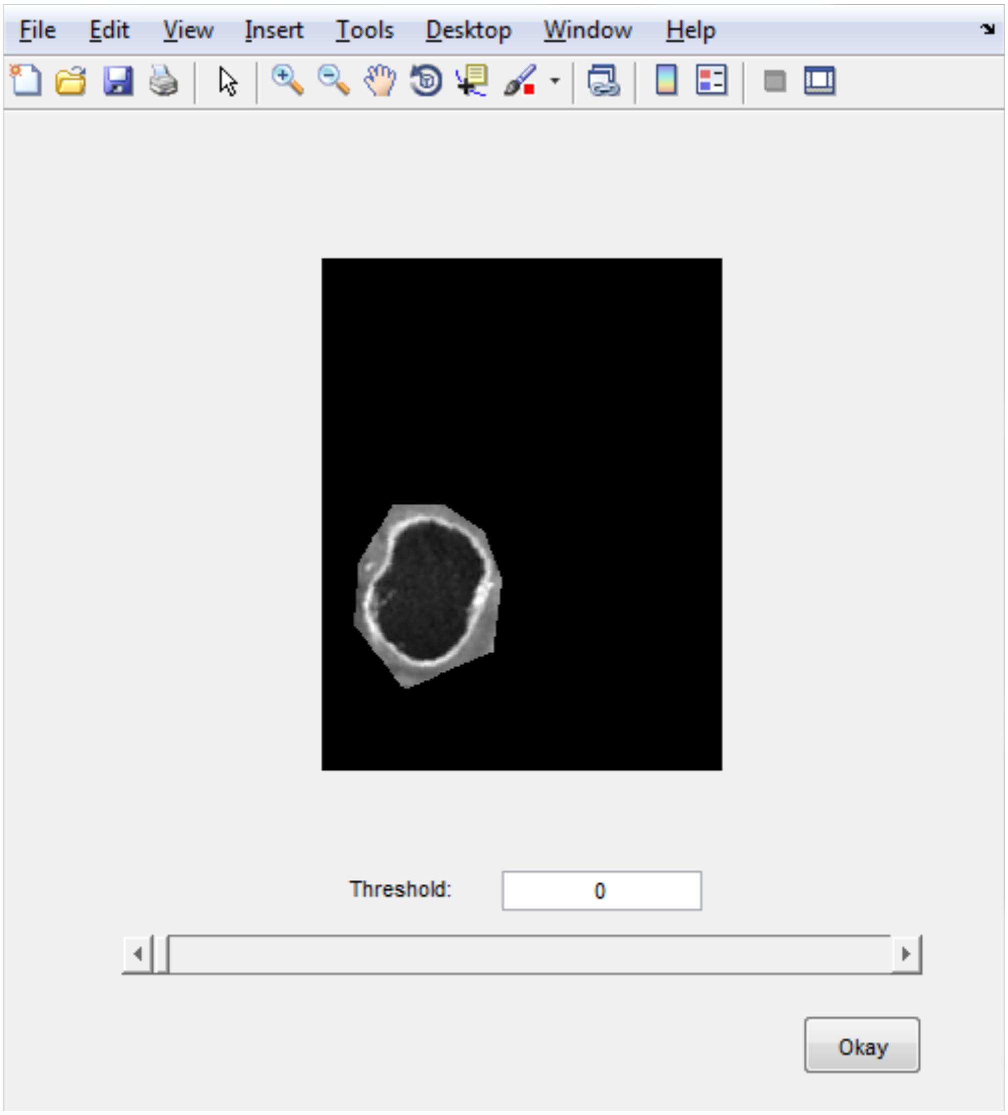
File Edit View Insert Tools Desktop Window Help
Threshold:
0
Okay

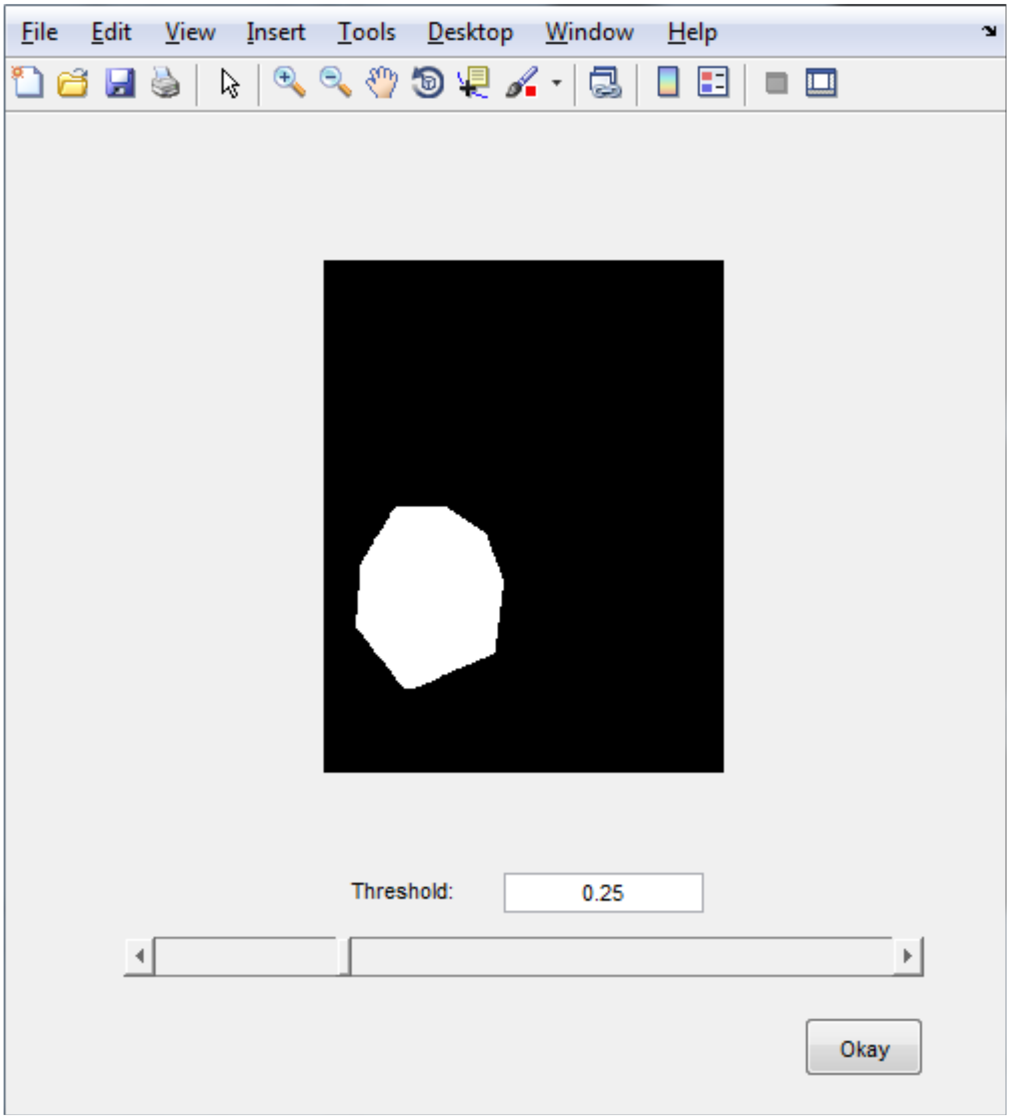
File Edit View Insert Tools Desktop Window Help
Threshold:
0.25
Okay

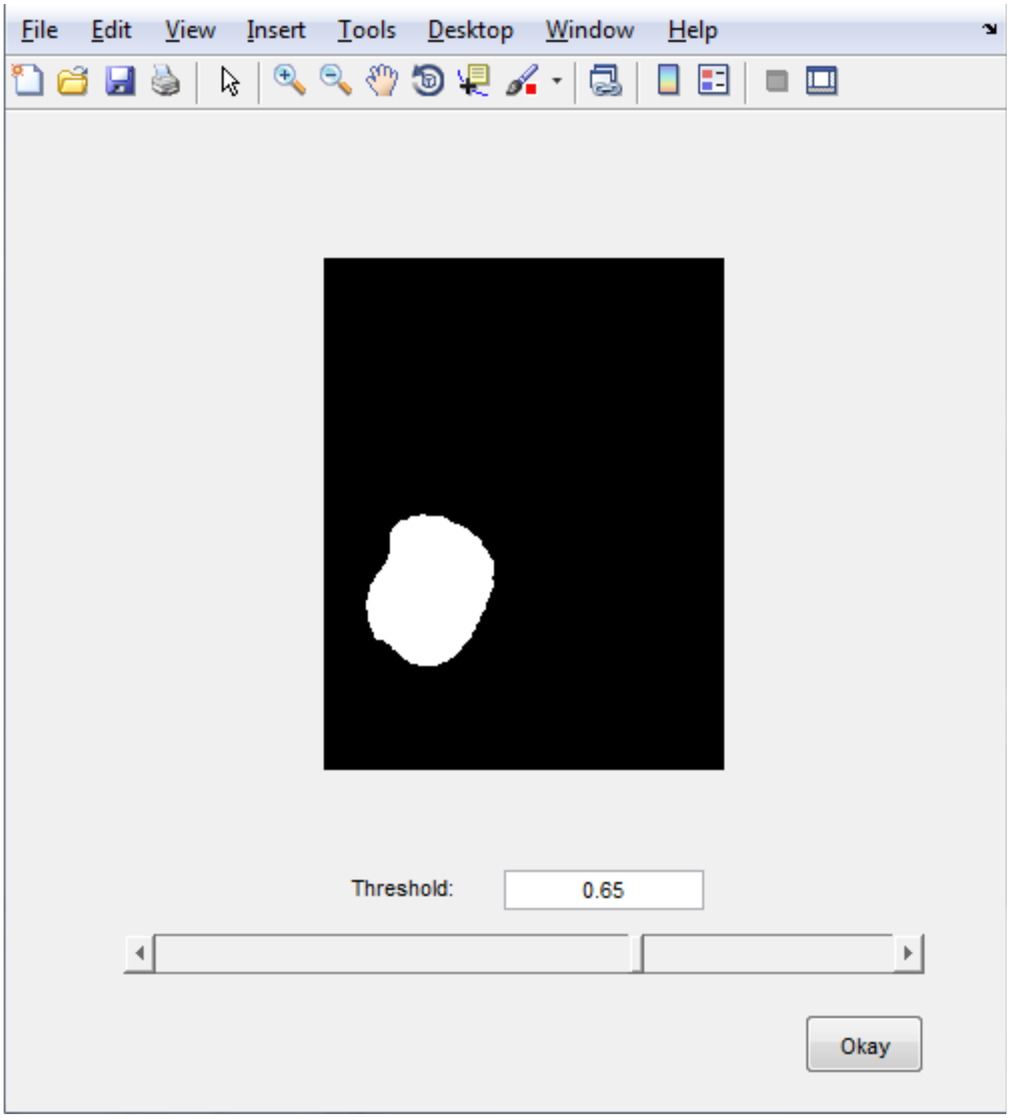
File Edit View Insert Tools Desktop Window Help
Threshold:
0.65
Okay

8. The tumor and peritumoral ring regions are then generated using a mask that restraints the ROIs from crossing into the contralateral hemisphere, the ventricles, and the skull.

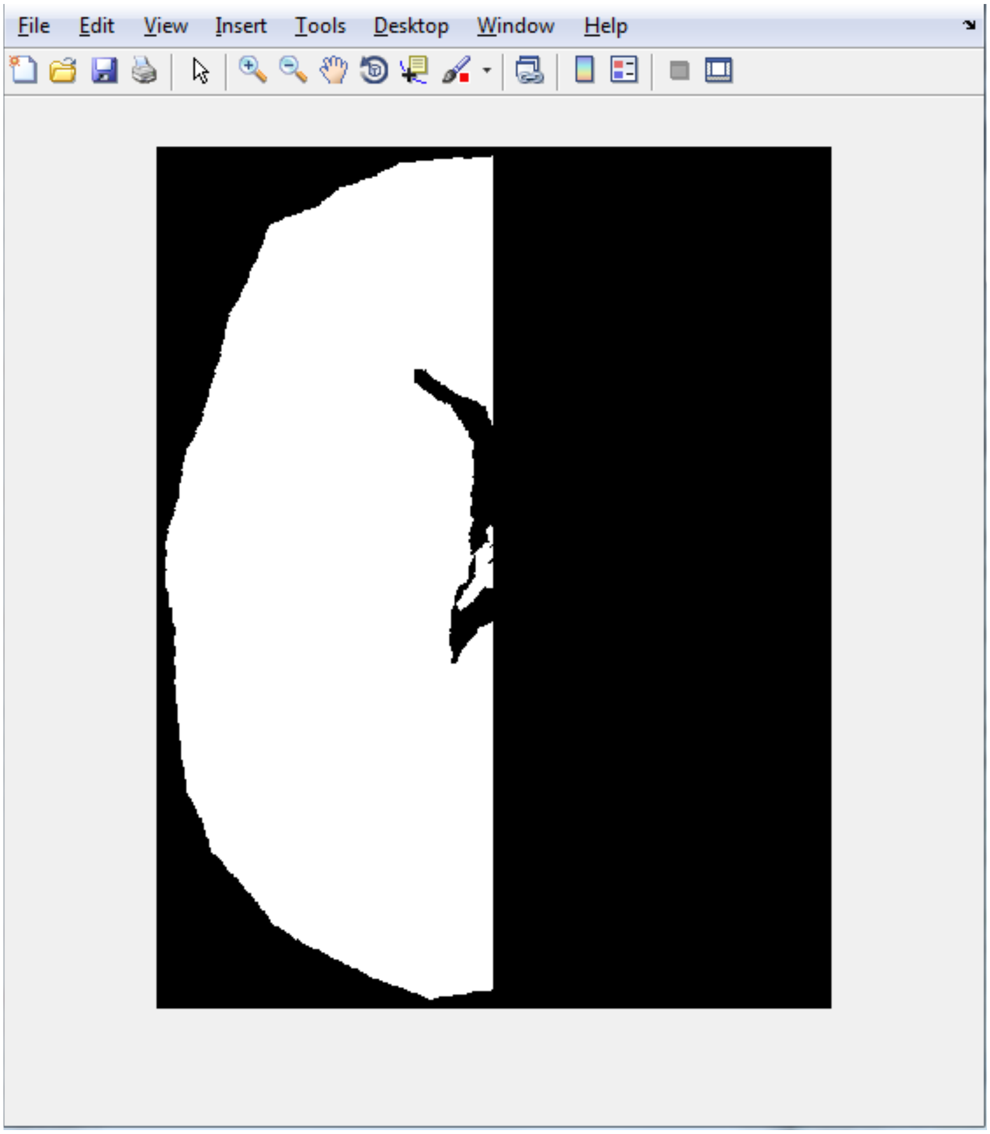

9. These binary masks are used to selected regions in the aligned FA, MD, and FLAIR image slices.

10. FA, MD, and FLAIR measures within the tumor and peritumoral region are then recorded and saved into spreadsheet output files.
